# The *Shh* Topological Domain Facilitates the Action of Remote Enhancers by Reducing the Effects of Genomic Distances

**DOI:** 10.1016/j.devcel.2016.10.015

**Published:** 2016-12-05

**Authors:** Orsolya Symmons, Leslie Pan, Silvia Remeseiro, Tugce Aktas, Felix Klein, Wolfgang Huber, François Spitz

**Affiliations:** 1 Developmental Biology Unit, European Molecular Biology Laboratory, Meyerhofstrasse 1, 69117 Heidelberg, Germany; 2 Genome Biology Unit, European Molecular Biology Laboratory, Meyerhofstrasse 1, 69117 Heidelberg, Germany

**Keywords:** gene expression, long-distance enhancers, chromatin conformation, Shh, topological domains

## Abstract

Gene expression often requires interaction between promoters and distant enhancers, which occur within the context of highly organized topologically associating domains (TADs). Using a series of engineered chromosomal rearrangements at the *Shh* locus, we carried out an extensive fine-scale characterization of the factors that govern the long-range regulatory interactions controlling *Shh* expression. We show that *Shh* enhancers act pervasively, yet not uniformly, throughout the TAD. Importantly, changing intra-TAD distances had no impact on *Shh* expression. In contrast, inversions disrupting the TAD altered global folding of the region and prevented regulatory contacts in a distance-dependent manner. Our data indicate that the *Shh* TAD promotes distance-independent contacts between distant regions that would otherwise interact only sporadically, enabling functional communication between them. In large genomes where genomic distances per se can limit regulatory interactions, this function of TADs could be as essential for gene expression as the formation of insulated neighborhoods.

## Highlights

• Potential for responsiveness to *Shh* enhancers correlates with contact frequency
• Changing distances within *Shh* TAD has little effect on *Shh* expression
• Inversions bridging the *Shh* TAD boundaries disrupt folding and regulatory contacts
• Decreasing genomic distance can rescue disrupted long-range regulatory interactions

## Graphical Abstract

**Figure:**
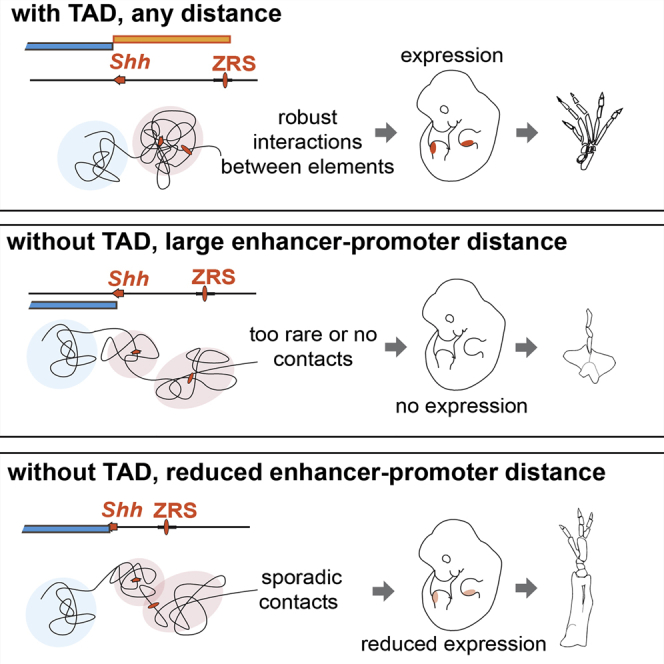

## Teaser

Using systematic genomic rearrangements at the *Shh* locus, Symmons et al. demonstrate that the topologically associating domain overlapping the locus acts as a functional regulatory mold by promoting contacts between elements that would otherwise be too rare to yield a functional outcome due to the dampening effects of large genomic distances.

## Introduction

A substantial fraction of gene regulatory elements lie at considerable distance from the nearest promoters (ENCODE Project Consortium et al., 2012, Shen et al., 2012, Visel et al., 2007). While the contribution of these elements to gene expression is generally difficult to estimate, enhancers located hundreds of kilobases from their target genes but essential to their expression are increasingly identified (Sagai et al., 2009, Sagai et al., 2005, Spitz et al., 2003, Uslu et al., 2014, Wunderle et al., 1998, Zuniga et al., 2004) (reviewed in de Laat and Duboule, 2013, Visel et al., 2009). Accordingly, mutations or genetic variants in distant enhancers are a significant cause of genetic diseases (Benko et al., 2009, Bhatia et al., 2013, D'haene et al., 2009, Lettice et al., 2003) and contribute to intra-species (Bauer et al., 2013, Smemo et al., 2014, Sur et al., 2012, Wasserman et al., 2010) and inter-species (Prescott et al., 2015, Prud'homme et al., 2007) phenotypic variability. Although our understanding of regulatory elements has improved tremendously in recent years, it remains unclear how enhancers find a specific target located several hundred kilobases away. There is strong evidence that such interactions require physical proximity (Deng et al., 2012). Yet, how this proximity is established and regulated and how it influences target gene expression is still poorly understood.

Concomitant with the growing appreciation of distant regulatory sequences, improved chromosome conformation capture techniques have provided insights into the three-dimensional organization of the genome and *cis-*interaction networks between genes and surrounding elements (Hughes et al., 2014, Kieffer-Kwon et al., 2013, Li et al., 2012, Mifsud et al., 2015). These approaches have revealed not only loops between distant elements but also that mammalian genomes are partitioned into sub-megabase-sized domains referred to as topologically associating domains or TADs (Dixon et al., 2012, Nora et al., 2012). Several indirect lines of evidence suggest that these self-interacting regions may represent the core units of genome regulatory architecture (Gibcus and Dekker, 2013); a large proportion of TAD boundaries are shared between cell types (Dixon et al., 2015) and largely preserved during evolution (Vietri Rudan et al., 2015). Coordinately regulated tissue-specific enhancer-promoter pairs (Shen et al., 2012) and associated long-range looping interactions (Dowen et al., 2014, Jin et al., 2013) are usually comprised within TADs. The regulatory domains defined by enhancers' range of action coincide also largely with TADs (Symmons et al., 2014). Although internal interactions within TADs can be cell-type specific and activity dependent (Phillips-Cremins et al., 2013, Rao et al., 2014), these different findings support the role of TADs as basic structural and functional units.

Correlations between regulatory and structural subdivisions of the genome suggest that TADs may constrain the range of action of enhancers, with TAD boundaries acting as functional “insulators” (Chetverina et al., 2013, Yang and Corces, 2012). TAD boundaries are indeed enriched for elements shown to have insulator activity (such as CTCF binding sites and transcriptional start sites) (Dixon et al., 2012), and insertions of a sensor gene on opposing sides of TAD boundaries show distinct expression patterns (Symmons et al., 2014, Tsujimura et al., 2015). Recent experiments deleting or altering these boundaries showed expansion of chromosomal contacts across the former boundaries, leading to ectopic activation of neighboring genes (Dowen et al., 2014, Lupiáñez et al., 2015, Narendra et al., 2015, Tsujimura et al., 2015). Similarly, the consequences of multiple human pathological chromosomal rearrangements can be explained by modification of TAD boundary positions and subsequent enhancer adoption by non-target genes (Flavahan et al., 2015, Hnisz et al., 2016). Together, these experiments clearly established that TAD boundaries are essential for generating isolated domains of regulatory activities. However, other features and potential roles of TADs remain poorly studied.

The *Shh* locus constitutes an ideal system to study long-range enhancer-promoter regulation. *Shh* expression is regulated by a series of tissue-specific enhancers distributed across a region spanning over 900 kb, which also comprises other unrelated genes (Jeong et al., 2006, Lettice et al., 2003, Sagai et al., 2009) and which corresponds to a tissue-invariant and evolutionary conserved TAD (Dixon et al., 2012, Jin et al., 2013). In particular, the specific expression of *Shh* in the zone of polarizing activity (ZPA), which establishes antero-posterior patterning of the developing limbs, is fully determined by the activity of a single *cis-*acting enhancer (Jeong et al., 2006, Lettice et al., 2003, Sagai et al., 2005). This element, the ZRS, lies 850 kilobases away from the *Shh* promoter, in an intron of an unrelated gene, *Lmbr1* (Lettice et al., 2003). In the present work, we took advantage of this prototypic enhancer-promoter pair to study the relationship between distant enhancer-promoter interactions, 3D conformation, and gene expression. We generated a series of mouse strains carrying tagged and structurally rearranged alleles of this locus. We analyzed them in vivo, when the mechanisms associated with its regulation are functional and biologically relevant, and in situ, in the genomic context where they evolved and normally operate. Our results showed that enhancer-promoter loops occurred within the framework of much more promiscuous contacts, where enhancers scan the entire topological domain they are part of. Remarkably, altering enhancer-promoter distances in the context of the *Shh* TAD did not appear to affect *Shh* expression. In contrast, disruption of the TAD prevented physical and regulatory interactions between *Shh* and its limb enhancer, unless the genomic distance between the two was significantly reduced. Our observations provide evidence that TADs ensure high contact frequency between distant elements by counteracting the effect of genomic distances. TADs do not simply restrict enhancer activity to a specific region to prevent ectopic interactions. They also provide the spatial proximity that is essential for efficient action of remote enhancers on genes located within the same TAD. This regulatory role of TADs can be particularly important in large genomes and may have enabled expansion of the genomic space available for regulatory innovation during evolution.

## Results

### The *Shh* Regulatory Domain: Extended but Variable Responsiveness to Enhancers

**Figure 1:**
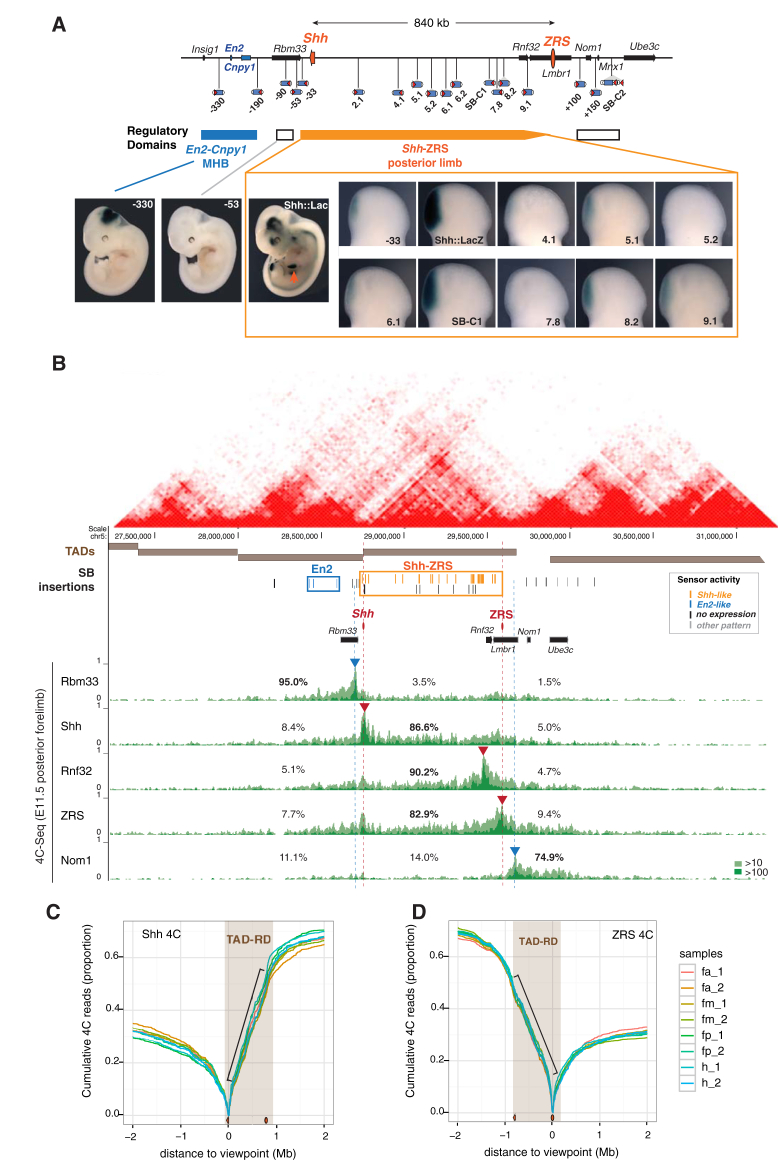
Topological and Regulatory Organization of the *Shh* Locus (A) Schematic representation of the *Shh* regulatory domain, as defined by the collection of 60 transposon insertions obtained with GROMIT. The location of representative insertions and their expression patterns is shown. Bars represent regulatory domains (*En2-Cnpy1*, *Shh*), as outlined by expression patterns reminiscent of the ones of the associated endogenous genes. White bars indicate that insertions in those regions have no expression. Orange arrowhead indicates the ZPA. (B) The *Shh* regulatory domain compared with the 3D conformation of the locus. Hi-C map of the locus from CH12 cells (Rao et al., 2014) (red contact maps, image generated with 3Dgenome browser, http://www.3dgenome.org) and TADs identified in ESCs (Dixon et al., 2012) (brown bars) are shown. Position and activity of insertions are indicated by colored lines (orange, *Shh*-like expression; blue, *En2-*like expression; black, no expression; gray, other/non-attributed expression). Corresponding regulatory domains are boxed. Shown beneath are 4C-interaction profiles (hit percentage with 10 and 100 count thresholds in light and dark green, respectively) of three viewpoints (*Shh*, *Rnf32*, ZRS, red arrowheads and lines) located in the regulatory domain and of two viewpoints (*Rbm33*, *Nom1*, blue arrowheads and lines) flanking it. For each viewpoint, we indicate the percentage of reads from regions in the *Shh* domain or from the 1 Mb flanking regions. (C and D) Cumulative 4C read counts as a function of distance from the *Shh* viewpoint (C) or the ZRS viewpoint (D). Data from different microdissected limb compartments is shown in different colors (fa, anterior forelimb; fm, medial forelimb; fp, posterior forelimb; h, hindlimb, 1 and 2 indicate biological replicates), the TAD/regulatory domain is highlighted in brown and the black bar indicates the constant slope of the curve.

We had previously shown that insertions of a regulatory sensor (Ruf et al., 2011) at the *Shh* locus show a *Shh-*like expression pattern, and reveal a large regulatory domain that overlaps with the TAD at this locus (Symmons et al., 2014). To get further information on how *Shh* enhancers act within this domain, we generated additional insertions of the regulatory sensor and analyzed its expression in mouse embryos at stages E10–12 (Figures 1A and S1, Table S1). The comparison of the patterns observed with 59 different insertions across the *Shh* genomic region provides a fine-scale view of its regulatory architecture, extending our first observations (Symmons et al., 2014) and those performed with a different promoter (Anderson et al., 2014). Noteworthy, like at other loci (Ruf et al., 2011), the insertion of this naive sensor did not alter the expression of *Shh* or surrounding genes (Figures S1C and S1D), indicating that the activity of the sensor does not trap enhancers away from *Shh*. Instead, the sensor reveals the pre-existing potential of surrounding enhancers to act on a given genomic position.

We found that in the region beginning 33 kb downstream of *Shh* and extending to the ZRS, most insertions showed expression patterns that closely matched *Shh* expression in the limb (Figure 1A) as well as in other tissues (Figure S1). Outside of this *Shh* regulatory domain, insertions showed no expression or a divergent one. The *Shh* expression patterns detected by insertions in the *Shh* regulatory domain included domains for which enhancers have been mapped (Jeong et al., 2006, Sagai et al., 2009) (Figure S1), as well as domains for which no enhancers have been identified to date (e.g., choroid plexus; Figure S1G). This widespread responsiveness indicated that most *Shh* enhancers can act long range and not only in their vicinity or close to the *Shh* promoter. We found that expression of the sensor at a given position was highly reproducible, both when comparing littermate embryos and in independent replicate experiments (Table S1). Yet, lacZ stainings of neighboring insertions can sometimes differ extensively, even when only a few kilobases apart (Figures 1A and S1). A small number of insertions within the regulatory domain, such as insertion 5.2, showed no expression in any tissue. But more typically, variation was quantitative and differed depending on the tissue. For example, at position 5.1, we observed robust expression in the notochord and floor plate, but only weak staining in the limb and in the genital bud; at position C1, we observed the reverse relative intensities (Figures S1B, S1E and S1F). The reporter insertion at position −33 showed high responsiveness to the ZRS but not to other enhancers (Figure S1). We also observed this quantitative variability at stages other than E11.5 (Figures S1G and S1H). Our data show that, for the same promoter, the responsiveness to enhancer(s) can vary extensively within an otherwise largely permissive regulatory domain.

To understand what factors modulate responsiveness to regulatory inputs, we focused on the limb where *Shh* expression is determined by a single enhancer, in contrast to many other tissues where it is associated with several enhancers with overlapping activities (Jeong et al., 2006, Sagai et al., 2009, Tsukiji et al., 2014). Critically, similarly to other tissues, the sensor showed significant variability in ZPA expression at different positions within the *Shh* TAD (Table S2). This indicated that variability in responsiveness is not limited to complex situations involving multiple enhancers. Responsiveness to the ZRS showed no correlation with linear distance to the ZRS nor did it appear to be influenced by the orientation of the sensor (Fisher exact test p = 0.387) or local chromatin features (proximity to repeat elements such as LINEs or SINEs; accessibility measured by DNaseI hypersensitivity or chromatin acetylation/methylation) (Table S2, data not shown) signifying that it is determined by other factors.

### Regulatory and Topological Domains Coincide at the *Shh* Locus

As noted before (Anderson et al., 2014, Symmons et al., 2014), the *Shh* regulatory domain shows strong overlap with an underlying TAD, conserved in different cell lines (Dixon et al., 2012, Jin et al., 2013, Rao et al., 2014) (Figure 1B). Since 3D conformations can vary between cell types, we performed chromosome conformation capture sequencing experiments (4C-seq) on the posterior compartment of E11.5 microdissected limb buds (Figures 1B and S2). We chose viewpoints within the TAD (*Shh* promoter, ZRS, and *Rnf32*) and outside (*Rbm33*, *Nom1*). In the posterior limb, the three viewpoints located in the *Shh* TAD showed prominent contacts along the entire TAD, while contact frequency with regions outside the TAD decreased quickly (Figure 1B). Reciprocally, the viewpoints located immediately outside the TAD, on either side, showed limited interactions with sequences in the *Shh* TAD (Figure 1B). Both the ZRS and *Shh* had contact frequencies that remained constantly high throughout the TAD, with limited effect of genomic distance (Figures 1C and 1D). Together, these data show that the *Shh-*ZRS region forms a self-interacting chromatin domain in the posterior limb bud, which corresponds well to the TAD described in other cell types.

### The Potential for Responsiveness Is Influenced by the 3D Organization

**Figure 2:**
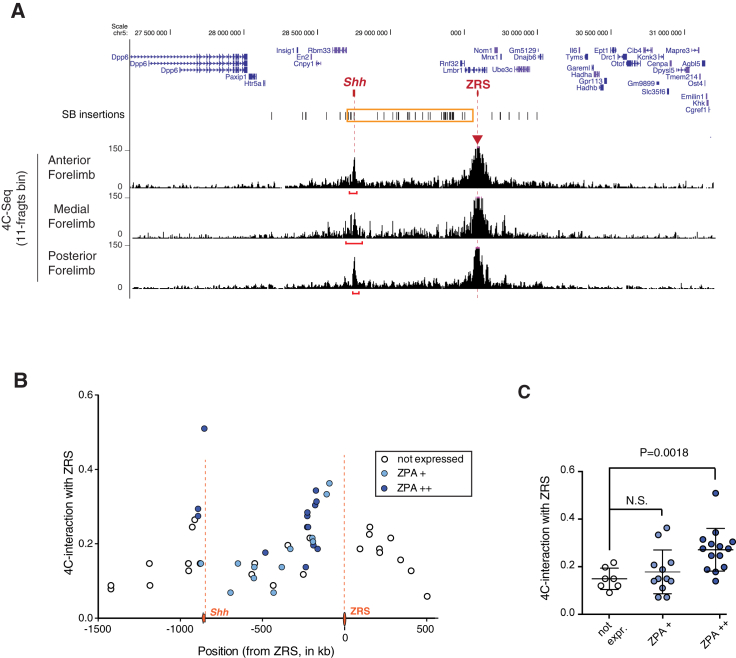
Organization of the *Shh* Locus and Responsiveness to ZRS (A) 4C-seq interaction profile (read counts, binned in 11-fragment sliding windows) of the ZRS (viewpoint indicated by red triangle) in the anterior, medial, and posterior compartments of E11.5 forelimbs. A red bar underlines the peak contact region around *Shh*. For further comparisons, see also Figure S2. (B) ZRS-interaction values at the insertion points of the transposon (in the absence of the transposon). x axis, distance to the ZRS; y axis, 4C-interaction score; dot color represents intensity of LacZ staining in the ZPA. (C) Comparison of interaction scores with responsiveness to the ZRS for positions within the *Shh* TAD (not expressed versus strongly expressed in ZPA; p = 0.0018, two-sided Mann-Whitney test).

Even among the generally robust interactions detected along the TAD, the ZRS showed a particularly stronger interaction with the *Shh* promoter in the posterior limb bud (Figure 2A), in agreement with previous 3C and FISH data (Amano et al., 2009). Interestingly, based on 4C-seq, the compartmentalization of interactions and the fine-scale interactions of the ZRS did not appear very different between E11.5 posterior limbs (where *Shh* and the ZRS are active) and E11.5 anterior and medial forelimb samples (where *Shh* and the ZRS are inactive) (Figures 1C, 1D, and S2); the ZRS showed stronger contact with *Shh* in all limb compartments (Figure 2A), although the interaction peaks appeared more diffuse in the inactive situations than in the ZPA. Hence, similarly to other loci (Ghavi-Helm et al., 2014, Montavon et al., 2011), conformation and enhancer-target gene contacts appear to be in part constitutive and independent of transcriptional and regulatory activity.

To understand if responsiveness of the regulatory sensor to the ZRS was related to the native folding of the locus, we compared the interaction profile of the ZRS (from wild-type limb, without sensor insertions) to the expression of the sensor in the ZPA at the different insertion sites (Figures 2B and Table S2). We found that, within the *Shh* TAD, positions with ZPA expression had overall stronger contacts with the ZRS than weakly or non-expressed ones (Figure 2C). This correlation indicates a relationship between the distribution of enhancer activity and the native structural folding of the locus. It should be noted, however, that some positions contacted by the ZRS with similar efficiency (as measured by 4C, at a resolution of 5–10 kb) displayed different activation potential, indicating that average contact frequency is not the sole determinant for regulatory activation.

### TADs Buffer the Effect of Genomic Distances between Promoters and Enhancers

**Figure 3:**
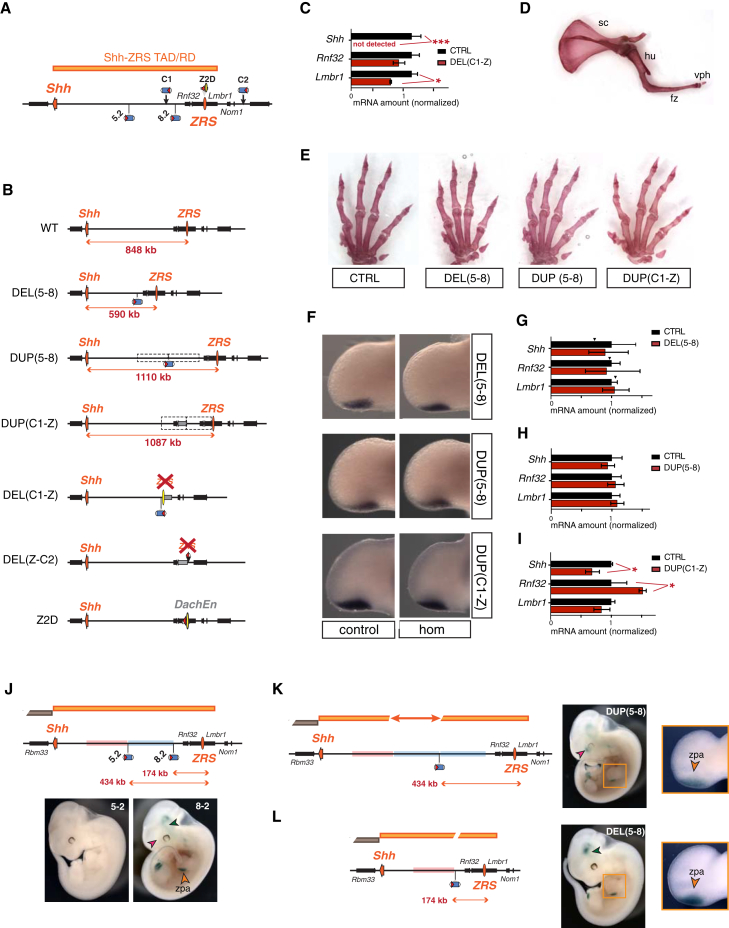
Changing Distances within the *Shh* TAD (A) Schematic representation of the region, including the different insertion points and *loxP* sites used. (B) Schematic representation of the rearranged alleles. The distance separating the ZRS (orange oval) from *Shh* is indicated. The transposon at the junction point (when retained) is indicated, and dashed rectangles mark the duplicated regions. The Z2D allele is a replacement of the ZRS by another limb enhancer (yellow oval, DachEn/hs126; Visel et al., 2007), which appeared to be essentially inactive when inserted at this position (Figure S3G). (C) Gene expression by RT-qPCR in DEL(C1-Z) versus WT E11 forelimb buds (for each gene, reference value in WT set as 1, the error bars correspond to SEM. Statistical significance done with *t* tests: ^∗^p < 0.05; ^∗∗∗^p < 0.001. (D) Forelimb skeleton of a DEL(Z-C2)/*Shh*^*del*^ mouse showing monodactyly and fused zeugopod. sc, scapula; hu, humerus; fz, fused zeugopod; vph, vestigial phalanges. (E) Hand skeletons of adult mice with different rearranged alleles. Alleles are in *trans* of either *Shh*^*del*^ (for DUP(5–8) and DUP(C1-Z)) or of a ZRS replacement (Z2D allele, for CTRL and DEL(5–8)), because DEL(5–8) homozygous or compound mutants with *Shh*^*del*^ die at birth due to holoprosencephaly and cranial defect (not shown). (F) Expression of *Shh* in E10.5 forelimbs in the different alleles. For each line, in situ hybridization was performed on wild-type control littermates and homozygous mutants (n = 3). (G–I) RT-qPCR data in DEL(5–8) (G), DUP(5–8) (H), and DUP(C1-Z) (I) E11 forelimb buds. Homozygous mutant samples are in red (n = 3), stage-matched wild-type samples from the same litters (n = 3) are used as control, except for (G), where wild-type samples include embryos from separate litters (the arrowheads indicate the expression level in wild-type littermates of the mutants). The error bars correspond to SEM. ^∗^p < 0.05 (t test). (J–L) LacZ staining of E11.5 embryos with insertions of the sensor at positions 5.2, 8.2, and in the context of DEL(5–8) and DUP(5–8). On the schematic representation of the alleles, the *Shh-*ZRS TAD is in orange, and red and blue rectangles label the centromeric and telomeric flanking regions of 5.2, respectively. The sensor showed expression in the ZPA in 8.2 DUP(5–8) and DEL(5–8) embryos (orange arrowheads and insets) but not in 5.2 embryos. Expression domains observed in DUP(5–8) or DEL(5–8), but in none of the starting insertions, are labeled with pink and green arrowheads, respectively. For further gene expression and phenotypic data. See also Figure S3.

To further identify the mechanisms that govern distant interactions, we decided to systematically change different genomic parameters of the locus. First, we modulated the genomic distance separating the ZRS from *Shh*, while respecting the TAD boundaries. To this end, we engineered mice carrying either intra-TAD deletions or duplications, using *Cre-loxP* in vivo recombineering (Hérault et al., 1998) (Figures 3A and 3B). We then assessed limb morphology and *Shh* expression in animals carrying these rearrangements over a *Shh* null allele (*Shh*^*del*^) (Gonzalez-Reyes et al., 2012).

In agreement with ZRS deletion alleles (Lettice et al., 2014, Sagai et al., 2005), deletions that included the ZRS completely abolished limb expression of *Shh* (Figures 3C and S3A) and led to fore- and hindlimb monodactyly (Figures 3D and S3). We also observed loss of limb expression of the inserted sensor gene retained in DEL(C1-Z), showing that the remaining region comprised no limb enhancer. In contrast, compound embryos carrying either the DEL(5–8) deletion, which reduced the distance between *Shh* and the ZRS by 260 kb, or duplications that increased the distance to 1.1 Mb, DUP(5–8), DUP(C1-Z), showed normal limb morphology (Figures 3E and S3G). We did not detect major changes in *Shh* expression in E10.5 forelimbs as assessed by in situ hybridization (Figure 3F) and RT-qPCR (Figures 3G–3I, S3D, and S3F). We observed a slight reduction of *Shh* expression with the DUP(C1-Z) allele (*Shh-*ZRS distance = 1.08 Mb), but since the other duplication of similar size, DUP(5–8) (*Shh-*ZRS distance = 1.11 Mb), showed normal levels of *Shh*, this effect cannot be due solely to the increased distance. The difference between the two duplications may stem from the extra copy of *Rnf32* in DUP(C1-Z), which could act as a competitor for ZRS activity. However, previous reports have shown that *Rnf32* is not regulated by the ZRS (Amano et al., 2009), and we did not detect upregulation of *Rnf32* (Figure 3I) beyond the 1.5-fold increase that corresponds to the increase in *Rnf32* copy number from 2 in *Shh*^*del/+*^ to 3 in *Shh*^*del*^/DUP(1C-Z).

Altogether, *Shh* expression appeared largely resilient to changes in enhancer-promoter distances when TAD boundaries were left unchanged and when no element normally external to the *Shh* TAD was introduced.

### TAD Content Influences the Distribution of Enhancer Responsiveness

We next examined if these intra-TAD rearrangements, which showed no major impact on *Shh* expression, could nonetheless alter the distribution of ZRS responsiveness. Genes outside the *Shh* TAD *(Lmbr1*, *Rbm33* or *Nom1*) showed no significant expression changes in any of these genomic configurations (Figure S3), showing that confinement of enhancer activity is maintained. To look at the responsiveness within the TAD, we compared the expression of the regulatory sensor in the native context and in the context of the genomic rearrangements (Figure 3B). Prior to rearrangement, position 5.2 is refractory to activation by *Shh* enhancers, constituting one of the rare “dead spots” present in the domain, while 8.2 responds to multiple *Shh* enhancers (Figure 3J). Surprisingly, in the context of DUP(5–8), the sensor showed robust expression in the ZPA (Figure 3K), even though its position is identical to 5.2 with respect to the ZRS (same distance, same intervening sequences). Moreover, additional *Shh* expression domains (not observed at positions 5.2 and 8.2, but detected with insertions elsewhere in the locus), were also un-masked in the context of DUP(5–8) and DEL(5–8) (Figures 3K and 3L, pink and green arrowheads). Some of the new expression domains in DUP(5–8) may be associated with duplicated enhancers. But as the ZRS, the only limb enhancer active in the region, is located far outside the duplicated region, the gained expression in DUP(5–8) and in DEL(5–8) requires another explanation. We considered first that expression at position 5.2 could be locally repressed. If this is done by a centromeric repressor element, the reporter in DEL(5–8) should also be repressed. If this putative repressor was telomeric to position 5.2, then the reporter in DUP(5–8) should be repressed. Since both DEL(5–8) and DUP(5–8) show activity, the hypothesis of a local repressor at 5.2 is unlikely, as it would imply the existence of a cryptic de-repressor next to 8.2 that can counteract the repressor at 5.2. Even if we cannot fully rule out the existence of such a series of local elements, we propose that the rearrangements modulate the relative 3D folding of the TAD, and therefore change which regions are functionally exposed to the influence of the enhancers dispersed throughout this domain. This model is not only more parsimonious, but also fits well with the wide distribution of sensor cold spots, which correlates with 3D conformation.

### TAD-Breaking Inversions Disrupt Regulatory Interactions between *Shh* and the ZRS

**Figure 4:**
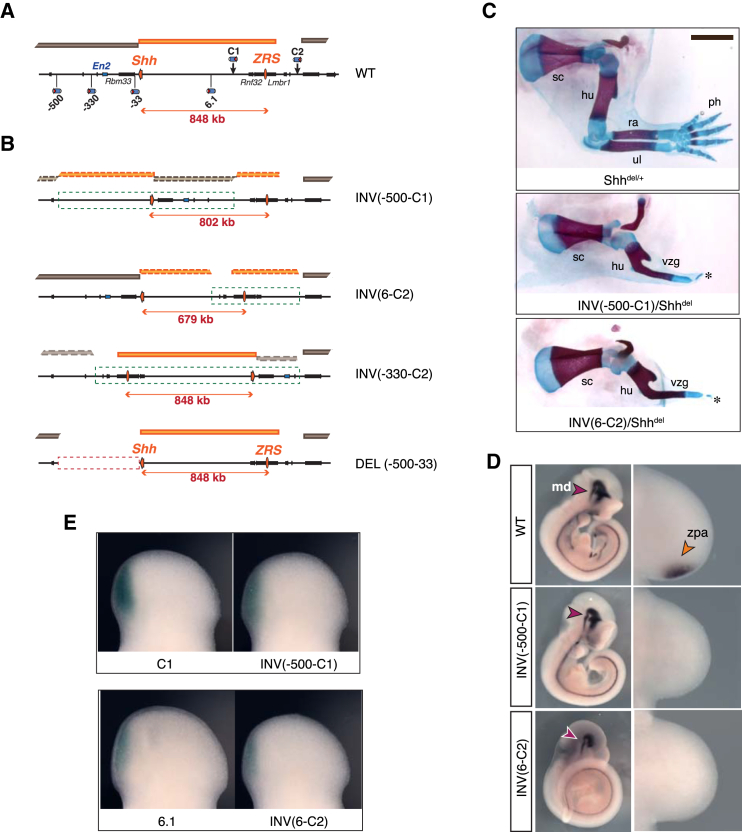
Consequences of TAD Disrupting Alleles (A) Representation of the insertions used to produce the inversions and del(-500-33). The *Shh*-ZRS TAD is in orange, the flanking ones in brown. (B) Representations of the rearranged alleles, with the inverted and deleted regions outlined by dashed green and red boxes, respectively. The linear distance between *Shh* and the ZRS is indicated. Dashed orange and brown blocs indicate the segment corresponding to former TADs. (C) Forelimb skeletons of E18.5 embryos for *Shh*^*del/+*^ (control), INV(-500-C1)/*Shh*^*del*^, and INV(6-C2)/*Shh*^*del*^, with the latter two showing the typical *Shh* loss of function limb phenotype. sc, scapula; hu, humerus; ra, radius; ul, ulna; vzg, fused zeugopod; ^∗^, single vestigial digit. (D) Expression of *Shh* in E10 mouse embryos by in situ hybridization. The orange arrowhead indicates expression in the ZPA, the purple one expression in the ventral midbrain (md). (E) LacZ expression in E11.5 autopods of embryos carrying the starting insertions in normal (C1, 6.1) or inverted configurations, INV(-500-C1), INV(6-C2). See also Figure S4.

The resilience of *Shh* expression to changes in enhancer-promoter distance can be interpreted as evidence for classical looping models, where *Shh* is directed and tethered to the ZRS. Such looping interactions could be driven by a combination of elements present at the enhancer (Lettice et al., 2014) or the promoter (Calhoun and Levine, 2003, Kwon et al., 2009, Williamson et al., 2011, Zabidi et al., 2015). To test these models, we engineered balanced inversions that should split the *Shh*-ZRS TAD, while keeping *Shh* within the range of action of the ZRS defined by the previous experiments (Figures 4A, 4B, and S4). INV(-500-C1) exchanged sequences between the *Shh* TAD and the centromeric *En2-Rbm33* TAD, while INV(6-C2) interspersed a region telomeric to the *Shh* TAD between the two halves of the original *Shh* TAD. In both cases, *Shh*-ZRS distances remained below 850 kb.

Animals carrying these inversions over a deletion of *Shh* (Figure 4C) or an inactivating substitution of the ZRS (Figures S4B and S4C) showed monodactyly on both fore- and hindlimbs. Expression of *Shh* was lost in the limb of E10 embryos homozygous for either inversion (Figure 4D). Importantly, *Shh* expression was detected in other tissues (Figure 4D), showing that the gene was not globally repressed. Furthermore, in both configurations, the associated regulatory sensor remained at the same position relative to the ZRS before and after inversion and maintained expression in the ZPA (Figures 4E, S4D, and S4F), indicating that the endogenous activity of the ZRS is unaltered. *Shh* loss of expression is also unlikely to result from the disruption of a specific accessory element, since the two inversions used different breakpoints. As further controls, we produced two additional rearrangements, INV(-330-C2) and DEL(-500-33), this time changing the sequences flanking the *Shh* TAD without modifying the TAD itself (Figure 4B). In both configurations, *Shh* expression and function appeared unaffected (Figures S4G and S4H), which led us to conclude that disruption of the neighboring domains had minimal effect on *Shh* regulation.

Overall, our experiments argue against the presence of a strong specific recognition system that will suffice to bring together *Shh* and the ZRS, as shown by the lack of *Shh* limb expression in INV(6-C2) and INV(-500-C1), despite shorter genomic distances than normal. These inversions, which reshuffled sections of different TADs, were the only ones from our series of rearrangements that affected *Shh*-ZRS communication, further strengthening the importance of TADs as regulatory units.

### TAD-Breaking Balanced Inversions Affect the Global Topology of the Locus

**Figure 5:**
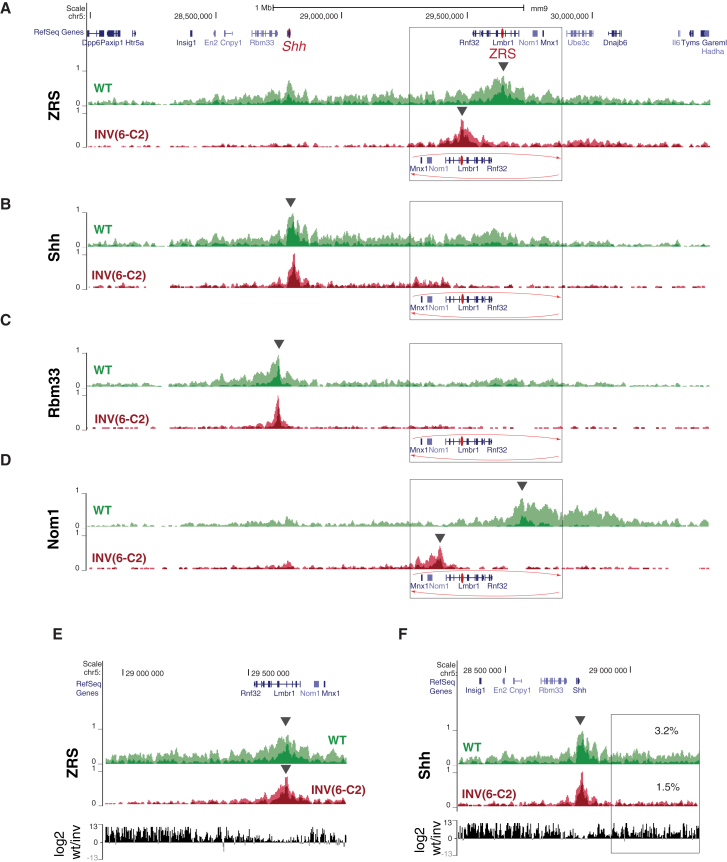
4C Profiles in INV(6-C2) Alleles (A–D) For each viewpoint, the hit percent profiles (with 10 and 100 count thresholds in light and dark color, respectively) obtained from WT (green) and INV (red) samples are plotted on their respective genomic configurations (i.e., with an inversion of the [6-C2] genomic segment for INV). The inverted region is boxed, and the new position of the genes in the INV allele is depicted. The viewpoints are indicated by black arrowheads. To take into account the loss of *Shh* expression and monodactyly in INV(6-C2), we compared INV whole forelimbs with WT anterior forelimb compartments. (E) Comparison of the interaction profile of the ZRS between WT and INV in the inverted region (plotted with the same orientation). (F) Same comparison as in (E) for the interaction profile of *Shh* between WT and INV. The box delimits the intra-TAD segment not affected by the inversion and the percentage of counts contained within it. See also Figure S5.

To assess the consequences of the TAD-reshuffling inversions on the topology of the locus, we repeated the 4C analysis in E11.5 limbs for the INV(6-C2) allele (Figures 5 and S5). To account for the loss of *Shh* expression following inversion, we compared INV(6-C2) forelimb 4C profiles with the ones obtained from the anterior compartment of E11.5 WT limbs. We found that the reciprocal interaction peaks between *Shh* and the ZRS found on the WT allele were lost in INV(6-C2) (Figures 5A and 5B). In addition, *Shh* and ZRS contacts became mostly local, focused in ∼100 kb around each viewpoint, replacing the broadly distributed contacts throughout the *Shh*-ZRS TAD characteristic of the wild-type chromosome (Figures 5E and 5F). This was particularly striking for the region between *Shh* and the inversion breakpoint, since the linear organization of this segment is not directly changed by the inversion (Figure 5B). In INV(6-C2), *Shh* showed some interactions with the *Mnx1*, *Nom1*, and *Lmbr1* promoters, but only marginally above what was observed in WT (Figures 5B and 5D), particularly if the reduced distance is considered. We did not observe broad reciprocal contacts between the *Lmbr1*-*Nom1* viewpoint and the *Shh* region, which could have indicated the reformation of a new TAD, as described at other loci (Lupiáñez et al., 2015, Tsujimura et al., 2015). Instead, in this case, the global reduction of contact frequency suggests that the overall conformation of the locus is disrupted, changing from a dense network of interactions along the TAD, either into a diffuse structure or into small, independent domains. Interestingly, the flanking viewpoints *Nom1* and *Rbm33* still displayed an asymmetric distribution of their contacts, avoiding the *Shh-*ZRS interval, indicating that some “insulating” aspects were retained (Figures 5C and 5D).

### Insulators versus Distance Effects

**Figure 6:**
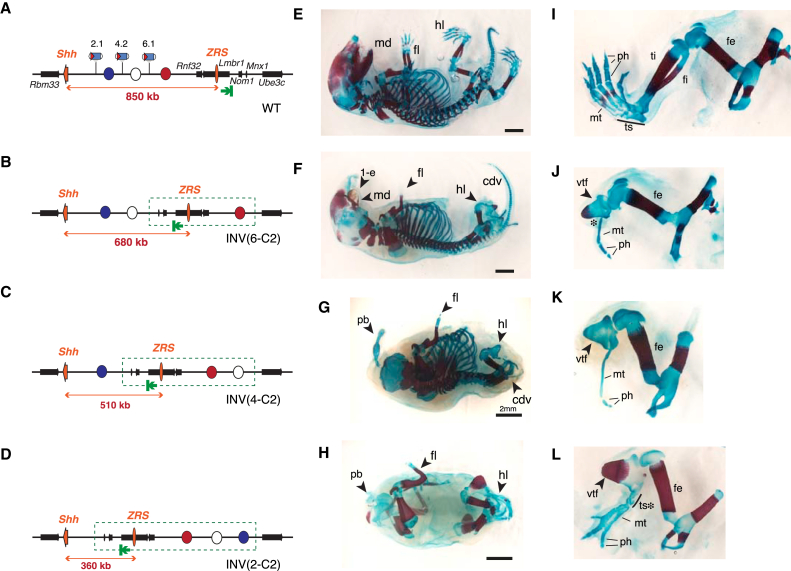
Distance-Dependent Rescue of ZRS Activity on *Shh* (A–D) Schematic representation of the series of inversions generated from C2. Red, blue, and white circles indicate putative enhancer elements that are progressively moved away from *Shh* by the inversions. A green arrow identifies the end of the *Shh* TAD. (E–H) Skeletons of E18 embryos, including close-up views of the hindlimb (I–L). Scale bar, 2 mm. (E and I) Control embryo (*Shh*^*del/+*^). (F and J) INV(6-C2)/*Shh*^*del*^. (G and K) INV(4-C2)/INV(4-C2). (H and L) INV(2-C2)/INV(2-C2). md, mandibule; fl, forelimb; hl, hindlimb, ph, phalanges; mt, metatarsal bones; ts, tarsal bones; ti, tibia; fi, fibula; cdv, caudal vertebrae. Arrowheads and asterisks point to deformed structures (cyclopia (1-e), vtf, vestigial partially fused tibia-fibula; pb, proboscis replacing anterior head structures). Photo in (E) was assembled from two images of the same embryo using Adobe Photoshop Photomerge script. See also Figure S6.

Our data from the INV(6-C2) and INV(-550-C1) inversions showed that gross disruption of the *Shh* domain abolished the ability of the ZRS to contact and activate *Shh*. This loss of contact could be explained by the presence of an insulator at the telomeric end of the TAD, since it would be relocated between *Shh* and the ZRS in the inversion. To test this possibility, we generated additional inversions, which utilized the same C2 telomeric breakpoint and different centromeric breakpoints (4.2 and 2.1), located closer to *Shh* (Figure 6A). The resulting rearranged alleles repositioned the same ZRS-*Lmbr1*-C2 intervening sequence between *Shh* and the ZRS as in INV(6-C2) but displaced increasing portions of the *Shh* regulatory domain (Figures 6B–6D). At first glance, as more enhancers were moved away, we observed a progressive increase in the severity of the phenotypes (Figures 6E–6H). This was particularly obvious for the cranio-facial and axial skeletons, which showed a stepwise increase in the extent of malformations. INV(2-C2) mice essentially copied the phenotype of complete *Shh* null lines (Chiang et al., 1996) or of mice where the entire regulatory region is removed (Niedermaier et al., 2005).

Importantly, these inversions also moved the ZRS progressively closer to *Shh*. While INV(6-C2) almost fully recapitulated the ZRS null limb phenotype (Figure 6J), we observed a gradual recovery of the limb structures, especially in the hindlimb. INV(4-C2) embryos still showed severely affected monodactylous limbs (Figure 6K), but INV(2-C2) embryos showed partially restored hindlimb morphology: feet usually comprised three digits, with an anterior big toe with two phalanges and two toes with three phalanges, while the tibia-fibula elements were distinct and only partially fused (Figure 6L). These limb phenotypes imply a gradual restoration of antero-posterior polarity and growth of zeugopod and autopod structures, consistent with a partial rescue of *Shh* activity. While we were unable to detect *Shh* expression in the limb of E10.5 embryos, prior work on other ZRS mutants has shown that reducing *Shh* expression to 10% of wild-type level results in a somewhat less severe hindlimb phenotype than the INV(2-C2) embryos (Lettice et al., 2014). Therefore, the INV(2-C2) phenotype is consistent with expression that is either extremely low or that occurs only during a very limited time period.

Compound mutants over an inactive ZRS allele (Z2D; Figure S6) also showed the same progressive restoration of limb morphology, indicating allelism to ZRS activity. In brief, this allelic series reveals that reducing *Shh-*ZRS distance can restore functional interactions between these elements, and that the presence of the *Lmbr1*-C2 region is not sufficient to block these interactions.

## Discussion

Although the ability of enhancers to act in a distance-independent manner is part of their original definition (Banerji et al., 1981), this property was established on plasmid assays (i.e., at distances up to 10 kb). In their native genomic environment, enhancers have been shown to select their target gene through mechanisms influenced by proximity (Dillon et al., 1997, Kmita et al., 2002), even though promoter preference (Butler and Kadonaga, 2001, Ohtsuki et al., 1998, Zabidi et al., 2015), occupancy by specific transcription factors (Deng et al., 2012), and/or tethering elements (Calhoun et al., 2002) may modulate these effects. Our present study of the *Shh* locus provides new insights into the organizing principles of long-distance enhancer-promoter interactions.

### Domain-wide but Variable Action of Remote Enhancers

**Figure 7:**
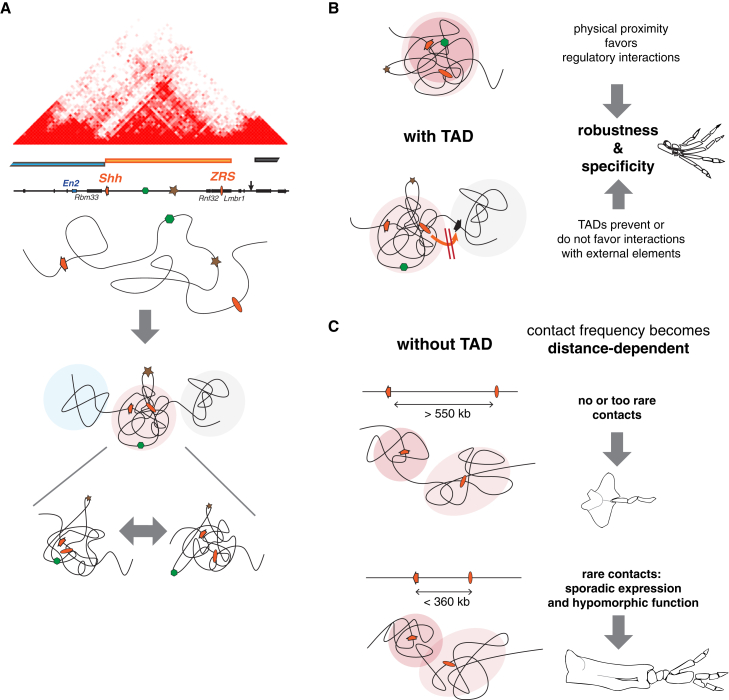
TADs Organize Robustness and Specificity of Long-Distance Interactions (A) TADs show an internal structure that determines the propensity of a region to be contacted by another one, hence defining enhancer responsiveness. *Shh* gene, orange arrow; ZRS, orange oval; an enhancer cold-spot, brown star; a responsive spot, green hexagon. The region folds into three different TADs (blue, orange, and dark gray bars), each of which likely corresponds to a dynamic ensemble of 3D conformations (below) (Fudenberg and Mirny, 2012, Giorgetti et al., 2014). The light colored area represents the region effectively explored by an element (e.g., the ZRS), i.e., with sufficient contact frequency to elicit a transcriptional response. The cold spot is located outside this zone, whereas the responsive spot can come in proximity of the ZRS. (B) TADs contribute to long-distance regulatory interactions by favoring proximity between otherwise distant regions (the two colored ovals represent the regions explored by *Shh* and the ZRS, respectively; the extent of overlap indicates frequent interactions). Elements located in distinct TADs do not influence genes located in the adjacent ones, not necessarily because of active insulation but simply because of the absence of a mechanism compensating for the buffering effect of genomic distances. (C) Without TADs, contacts between distant regions are too rare to be functional or lead only to sporadic gene activation producing variable phenotypic outcomes. See also Figure S7.

Confirming previous reports (Anderson et al., 2014, Symmons et al., 2014), our data demonstrate that enhancers act not only on their immediate neighborhood or on their target gene(s) but more generally across large domains. Our high-resolution characterization of the *Shh* regulatory domain highlights that the potential to respond to a given enhancer shows peaks and troughs throughout an otherwise largely permissive interval. This potential can be different, depending on the promoter; for example, insertions immediately adjacent to *Rnf32*, which does not respond to the ZRS, showed expression in the ZPA; inversely, some insertions next to *Shh* were inactive (which could be also due to competition). But we also uncovered substantial variation in expression between insertions of the same reporter, even when separated by only a few kilobases. This variation indicates that other factors than promoter sequence modulate responsiveness. We found a good correlation between the physical proximity to the ZRS, as measured by 4C, and the propensity to respond to its enhancer activity. This suggests that the *Shh* region folds in a pattern that acts as a mold for enhancer action (Figure 7). This framework is flexible, as it comprises only a few regions that are completely unresponsive (Figure 7A). The re-activation of unresponsive positions after internal rearrangements (DUP/DEL(5–8)) indicates that these positions are not necessarily locally repressed but simply excluded from contacting enhancers. Interestingly, some responsive positions showed contact frequencies that were as low as unresponsive regions, revealing either the influence of other factors or the limits of 4C to measure some interaction parameters (e.g., duration of contacts in the context of an ensemble of dynamic conformations) (Fudenberg and Mirny, 2012, Giorgetti et al., 2014).

### Tethers and Insulators?

Previous studies have suggested that sequences close to or within the ZRS may target it to *Shh* (Amano et al., 2009, Lettice et al., 2014), through the formation of a large loop (Williamson et al., 2011). Our ability to detect the action of the ZRS throughout the TAD with a reporter gene argues against the need for a specific promoter to respond to the ZRS. The inability of the ZRS to contact *Shh* in most inversions further shows that the ZRS cannot find *Shh* if it is not located in the same TAD, demonstrating the absence of a TAD-independent system targeting the ZRS to *Shh*.

Recent studies have substantiated models proposing that enhancers act within a space delineated by insulators (Dowen et al., 2014). The existence of insulators is widely supported by experimental evidence that identified short regions that can block enhancer-gene interactions (Hark et al., 2000, Lupiáñez et al., 2015, Tsujimura et al., 2015), and our data do not challenge the general existence and role of insulators. Yet, previous studies have also suggested that, even in the absence of specific insulators, certain loci show restricted enhancer-promoter interactions (Kokubu et al., 2009), questioning the universal necessity for insulators. Supporting this alternative view, many TAD boundaries appear not to be strict boundaries but correspond to a gradual effect, in terms of contact frequencies or blocking enhancer activities. At the *Shh* locus, the centromeric boundary between *Rbm33* and *Shh* appeared much more marked than the telomeric one between the *ZRS* and *Lmbr1*, both from a structural (based on 4C and Hi-C data) and a regulatory (changes in enhancer responsiveness) viewpoint, suggesting the telomeric boundary may be less robust (or organized). Modeling of insulator action has indicated their effect is largely distance insensitive (Doyle et al., 2014). The restoration of a functional ZRS*-Shh* interaction in the INV(C2) allele, when the distance separating *Shh* and the ZRS is reduced, therefore argues against the presence of a strict, well-defined insulator element. Interestingly, whereas *Lmbr1* is not responsive to the ZRS in mice, its ortholog in the more compact chicken genome shows distinct expression in the ZPA (Maas and Fallon, 2004). Based on our observations, we suggest that large genomic distances can act as a buffer for regulatory interactions, without the need to invoke the presence of specific insulators.

### Overcoming the Dampening Effect of Long Genomic Distances

According to simple polymer models, contact frequency should decline sharply with increasing distances. Yet, Hi-C data have revealed that, below 700 kb (approximately corresponding to the size of TADs), interactions occur more frequently than predicted, suggesting that loops and long-lived crosslinks may facilitate interactions at shorter scales (Doyle et al., 2014, Mateos-Langerak et al., 2009). A recent study modeling the *Igh* locus emphasized the importance of spatial confinement to establish interactions (Lucas et al., 2014) and proposed that this is the main determinant for enhancer-promoter communication. Our data also demonstrate that the interactions weaving the *Shh* TAD are necessary for efficient long-distance enhancer-promoter interactions; in the context of the *Shh* TAD, genomic distance has a minimal effect on enhancer-promoter interactions, whereas distance becomes a critical factor when this TAD is disrupted. TADs increase interaction frequency between elements and reduce the otherwise limiting effect of genomic distances. TADs can therefore actively extend the functional reach of enhancers to distantly located target genes. It will be important to see to what extent genes are dependent on this functionality of TADs or if other, independent mechanisms have also evolved to ensure proper long-range regulation.

### The Nature and Function of TADs: Loops and Compaction

The principles that lead to TAD formation are still debated (Barbieri et al., 2013, Dekker and Mirny, 2016), although mounting evidence suggests that loops between CTCF sites, possibly mediated by cohesin complexes, are involved (Merkenschlager and Odom, 2013, Zuin et al., 2014). The presence and relative orientation of CTCF sites at both ends of the *Shh* TAD (Figure S7) partially fit with recent CTCF-based models (de Wit et al., 2015, Guo et al., 2015). Yet, our observations also show noticeable deviations from what could be predicted from such models.

Firstly, some of our sensors integrated beyond the CTCF site separating *Rbm33* and *Shh* showed expression in the ZPA, implying that the ZRS is not blocked by this CTCF site or limited to a strictly defined CTCF loop. With respect to the predicted CTCF loops, the ZRS would be just outside the CTCF loop containing *Shh*, and the WT and INV configurations would be similar, whereas their functional outcome is strikingly distinct. In contrast, one would expected a more important effect in DUP(C1-Z), as the ZRS is now moved away from the potential CTCF-mediated loop containing *Shh*. Our functional data therefore underline that binding and orientation of CTCF are not sufficient to predict regulatory outcomes.

Beyond the underlying mechanism(s), the decisive factor governing enhancer-promoter functional interactions is the frequency of physical interactions between these elements. In this respect, the relative degree of insulation (which essentially is how TADs are identified) is far less important than the 3D volume of a TAD and its internal dynamics. Addition or removal of sequences normally present in the *Shh* domain have a small but noticeable impact on enhancer action, whereas interspersing external sequences into the *Shh* TAD, like with INV(6-C2), leads to a loss of compaction associated with the TAD and reduced long-range interactions. This shows that interaction frequency within a TAD may depend on its internal sequence or chromatin organization and not only on loops determined at its extremities.

Controlling regulatory interactions is an essential function of genomes, and current models have put a lot of emphasis on insulation (Dowen et al., 2014, Lupiáñez et al., 2015). In animals with more compact genomes, insulators may be critical to avoid unwanted interactions between close neighbors, which could explain why *Drosophila* evolved multiple types of insulators (Yang and Corces, 2012). But in animals with large genomes and large intergenic distances, genomic distance per se can often suffice to limit functional interactions. In these conditions, promoting long-range interactions becomes crucial to ensure robustness of a system that would otherwise depend on rare, stochastic collisions. Absence of such a mechanism would lead to phenotypic variability as illustrated by INV(2-C2) animals (Figure 7). In this view, the formation of compact genomic domains like TADs and the diverse mechanisms that ensure both robust and specific long-range regulatory interactions may have been essential to expand the genomic toolbox of evolution.

## Experimental Procedures

### Transgenic Mice

The founder ShhSB-C1, ShhSB-C2, and Z2D mice were generated by homologous recombination in E14 embryonic stem cells (ESCs). We inserted a Sleeping Beauty transposon that carries a *LacZ* reporter gene and a *loxP* site at chr5:29,413,901 for ShhSB-C1 and at position chr5:29,854,582 for ShhSB-C2. The ShhSB-C2 insert also contained a second *loxP* site outside the transposon. For Z2D, the ZRS enhancer (chr5:29,641,240-29,642,424) was substituted with a *Dach1* limb enhancer (chr14:97,485,490–97,486,674) (Visel et al., 2007). Remobilization of the SB transposon and mapping of new insertions was performed as described (Ruf et al., 2011). Targeted rearrangements were produced by in vivo recombineering (Hérault et al., 1998, Spitz et al., 2005). *Shh*^del^ mice carry a deletion of the second and third exon of *Shh*, produced by *Cre*-mediated recombination of the *Shh-nLZ* transgene (Gonzalez-Reyes et al., 2012). *Shh-nLZ* mice were kindly provided by Andreas Kottmann (Columbia University, New York) and are referred to as *Shh::LacZ* mice in this paper. All lines were maintained by breeding with C57BL/6J mice. Genomic positions are given for using the mm9/NCBI37 assembly. Mouse experiments were conducted in accordance with the guidelines in place at the European Molecular Biology Laboratory, as defined and overseen by its Institutional Animal Care and Use Committee, in accordance with the European Directive 2010/63/EU.

### LacZ Staining, Whole-Mount In Situ Hybridization, and Skeletal Preparation

LacZ staining, whole-mount in situ hybridization, and skeletal preparation were performed according to standard protocols. Full details are in the Supplemental Experimental Procedures.

### Quantitative RT-PCR

Total RNA was isolated from microdissected tissue embryos using a PureLink RNA Mini Kit (Invitrogen) with on-column DNase I treatment; 200 ng to 1 μg of isolated RNA was reverse transcribed with a ProtoScript M-MuLV First Strand cDNA Synthesis Kit (New England Biolabs) using oligo-dT as primer. qPCR was performed on an ABI7500 system with SYBR Green (Applied Biosystems), and analyzed using the ΔΔC_T_ method. For data normalization, *TBP, GusB*, or *Hif1* was used as the reference gene, and each condition was normalized to stage-matched littermate controls. Primers are listed in Table S3.

### 4C-Seq

4C libraries were generated from microdissected embryonic limb tissues following published protocols (Simonis et al., 2007, van de Werken et al., 2012b) (see Supplemental Experimental Procedures for details). 4C libraries were generated by PCR amplification with primers containing barcodes and Solexa adapters (see Table S3). Viewpoints were analyzed in duplicate and approximately 40 libraries were pooled per sequencing lane. All samples were subjected to 50 bp single-read sequencing using Illumina HiSeq 2000.

For the analysis of 4C libraries, FASTQ files were de-multiplexed based on barcode and viewpoint primer sequences, allowing no mismatch (first eight bases were used). Primer sequences were trimmed keeping the first restriction site, and de-multiplexed libraries were aligned to the mm9 reference genome using Bowtie version 1.0.0 (Langmead et al., 2009). Aligned reads were then mapped to an in silico *NlaIII*-digested mm9 genome in order to filter out non-informative reads. Only reads mapping to fragment ends in the correct orientation were kept and assigned to the corresponding fragment end. Fragment read counts correspond to the sum of the counts obtained for each of their extremities. We assessed the quality of the libraries by determining the percentage of mapped reads and the percentage of reads mapping in *cis* (intra-chromosomal reads relative to the viewpoint) for cross-linking and digestion efficiency (van de Werken et al., 2012a). All samples showed similar library quality based on these parameters (see Table S4). 4C-seq reads were filtered as described in Klein et al. (2015) and down-sampled to match the number of the library with the lowest read count. Interaction values with the viewpoint were calculated using two measures: normalized raw read counts smoothened across 11 fragments and a hit percent rate (Denholtz et al., 2013), including a minimal threshold. For the latter approach, we transformed the 4C signal to a binary value (0 or 1) for each fragment, depending on whether the normalized read count was below or above a certain threshold (e.g., over 1, 10, or 100 counts). Fragments that fulfill the threshold criteria are termed hits. We calculated the hit percentage in a given window (e.g., 25, 51, or 101 fragments) as an estimate of the contact frequency and reliability of a given fragment. We compared the effect of different thresholds and window size on the reproducibility of the signals obtained with biological replicates (see log2 ratios plots in Figure S2). For the experiments displayed here, parameters with a 51-fragment binning size (∼10–20 kb length) with a read count threshold of 10 showed robust and reproducible contact patterns across the region of interest and were therefore used.

For the analysis of 4C data from samples carrying a genomic inversion, we inverted the reference genome in silico between the breakpoint coordinates and removed the fragments containing the breakpoints. To estimate the asymmetry of the interaction profiles, we calculated cumulative count distributions on each side of the viewpoint by using the counts of the sub-sampled libraries. In this analysis, we disregarded the fragments located at a distance less than 10 kb from the viewpoint to reduce the strong influence of the most proximal fragments. Data have been deposited on ArrayExpress (E-MTAB-4980 ).

## Author Contributions

F.S. conceived the project and designed it with O.S. O.S., L.P, T.A., and S.R. performed the experiments. F.K. and W.H. performed computational analysis of 4C data. F.S., O.S., L.P., S.R., and T.A. analyzed the data. O.S. and F.S wrote the manuscript with input and comments from the other authors.

## Supplemental Information

Document S1. Supplemental Experimental Procedures, Figures S1–S7, and Table S4

Table S1. List of Insertions of the Reporter Sensor within and around the *Shh* Locus, Related to Figure 1

Table S2. Comparison of Responsiveness to the ZRS and Genomic Properties, Related to Figure 2

Table S3. List of Primer Sequences, Related to Experimental Procedures

Document S2. Article plus Supplemental Information
