# Supplementary material for: The Shh Topological Domain Facilitates the Action of Remote Enhancers by Reducing the Effects of Genomic Distances
Source: Dev Cell. 2016 Dec 5;39(5):529–43. doi: 10.1016/j.devcel.2016.10.015 (PMC5142843; doi:10.1016/j.devcel.2016.10.015)
Supplement: Document S1. Supplemental Experimental Procedures, Figures S1–S7, and Table S4 [file mmc1.pdf]

Developmental Cell, Volume *39*

Supplemental Information

The *Shh* Topological Domain Facilitates the Action of Remote Enhancers by Reducing the Effects of Genomic Distances

Orsolya Symmons, Leslie Pan, Silvia Remeseiro, Tugce Aktas, Felix Klein, Wolfgang Huber, and François Spitz

SUPPLEMENTARY MATERIALS

Inventory of Supplementary Materials.

Figure S1, related to Figure 1. Regulatory potential is non-uniformly distributed throughout the whole *Shh* locus

Figure S2, related to Figure 2. Topological organisation at the *Shh* locus in different limb compartments

Figure S3, related to Figure 3. Effects of changing distances within the *Shh* TAD.

Figure S4, related to Figure 4. Consequences of TAD disrupting-alleles on gene expression and phenotypes

Figure S5, related to Figure 5. 4C profiles in INV(6-C2) alleles.

Figure S6, related to Figure 6. Phenotypic consequences of a TAD-breaking inversion series.

Figure S7, related to Figure 7. Chromatin and CTCF sites organisation around the *Shh*-ZRS locus.

Table S1, related to Figure 1. List of the different insertions of the regulatory sensor.

Table S2, related to Figure 2. Responsiveness to the ZRS and genomic features of the different insertions.

Table S3, related to Experimental Procedures. List of primer sequences.

Table S4, related to Experimental Procedures. List and characteristics of 4C libraries

Supplementary Experimental Procedures

Supplementary References

Figure S1 (related to Figure 1).

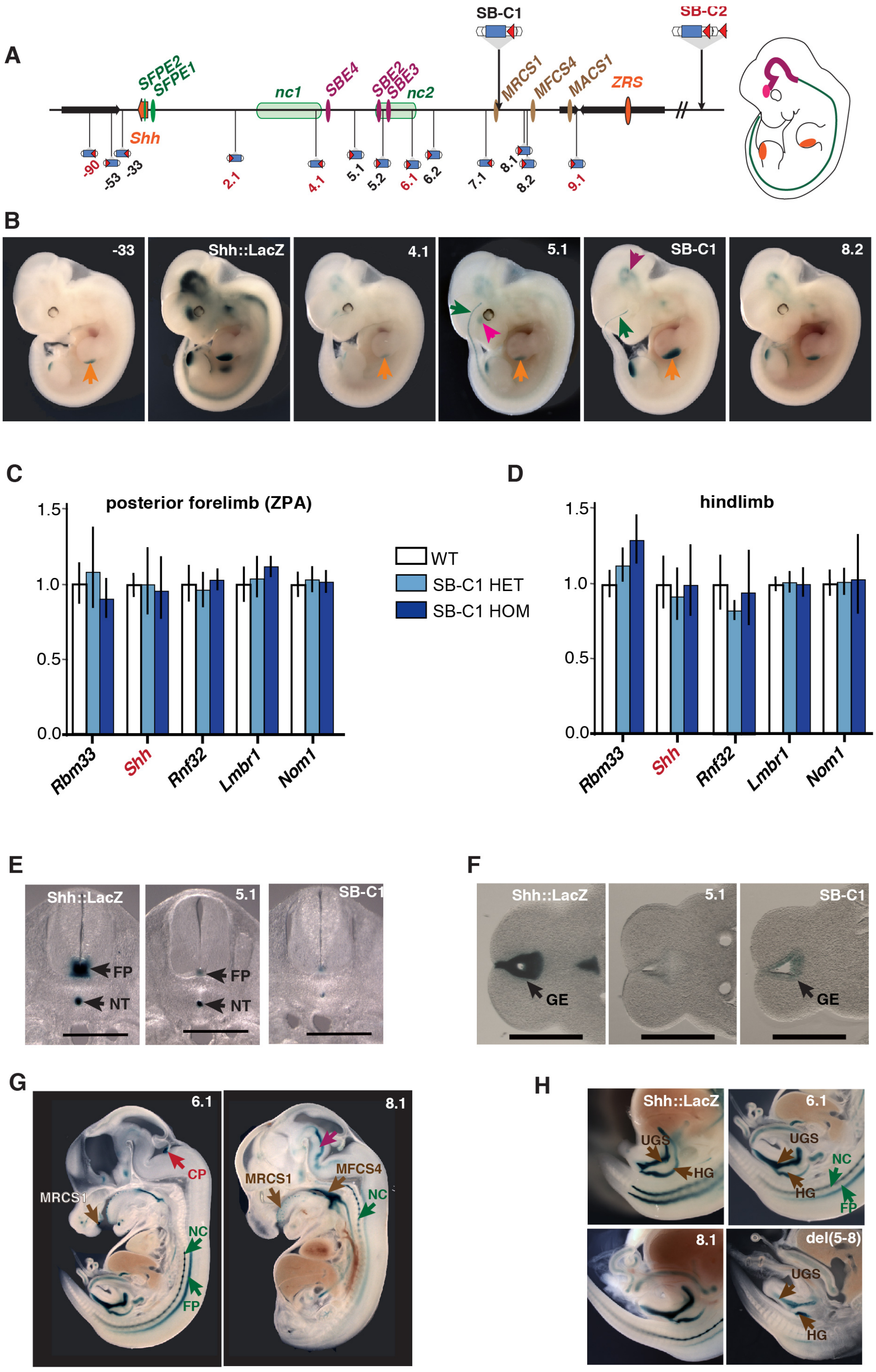

Figure S1, related to Figure 1. Regulatory potential is non-uniformly distributed throughout the whole *Shh* locus.

(A) *Shh* is regulated by an array of tissue-specific long-distance enhancers, which are distributed over a 900kb region. Their location along the locus and a schematic representation of their expression domains is shown, with overlapping/complementary enhancer activities indicated by matching colours. To capture the regulatory potential of the enhancers we inserted a transposable regulatory sensor at two different positions in the locus (indicated as SB-C1 and SB-C2). We subsequently remobilized the transposons and obtained multiple re-

integration events in the proximity of *Shh*. The positions of a subset of obtained insertions are shown below the locus. Black and red labels denote whether insertions were derived from SB-C1 or SB-C2, respectively. **(B)** Insertions at the locus capture the activity of different subsets of *Shh* enhancers, as revealed by lacZ staining of whole embryos. Most expression domains are observed throughout the locus, with most insertions. As examples, few domains are highlighted, with the colours of the arrows corresponding to the likely associated (orange: posterior limb buds/ZRS; green: notochord&floor plate/nc1/nc2/SFPE1/2; purple/pink: midbrain domains and ZLI. The lacZ pattern from a *Shh:lacZ* reporter line is shown as reference for the *Shh* expression pattern. **(C, D)** Gene expression in posterior forelimb (C) or hindlimb (D) of embryos heterozygous or homozygous for the SB-C1 insertion, as shown by quantitative RT-PCR. **(E-F)** Similar to the observations made on whole embryos, sections of lacZ-stained embryos also reveal non-uniform capture of regulatory activity **(G)** Differential capture of enhancer activity seen in E13.5 embryos. (**H**) Close-up view of the uro-genital region**.**
SFPE: *Shh* floor plate enhancer, SBE: *Shh* brain enhancer, nc: notochord, MRCS: Mammal-reptile conserved sequence, MACS: Mammal-amphibian conserved sequence, MFCS: Mammal-fish conserved sequence, ZRS: ZPA-regulatory sequence, FP: floorplate, GE: genital epithelium, UGS: urogenital sinus, HG: hindgut, CP: choroid plexus, NC: notochord.

Figure S2 (related to Figure 2).

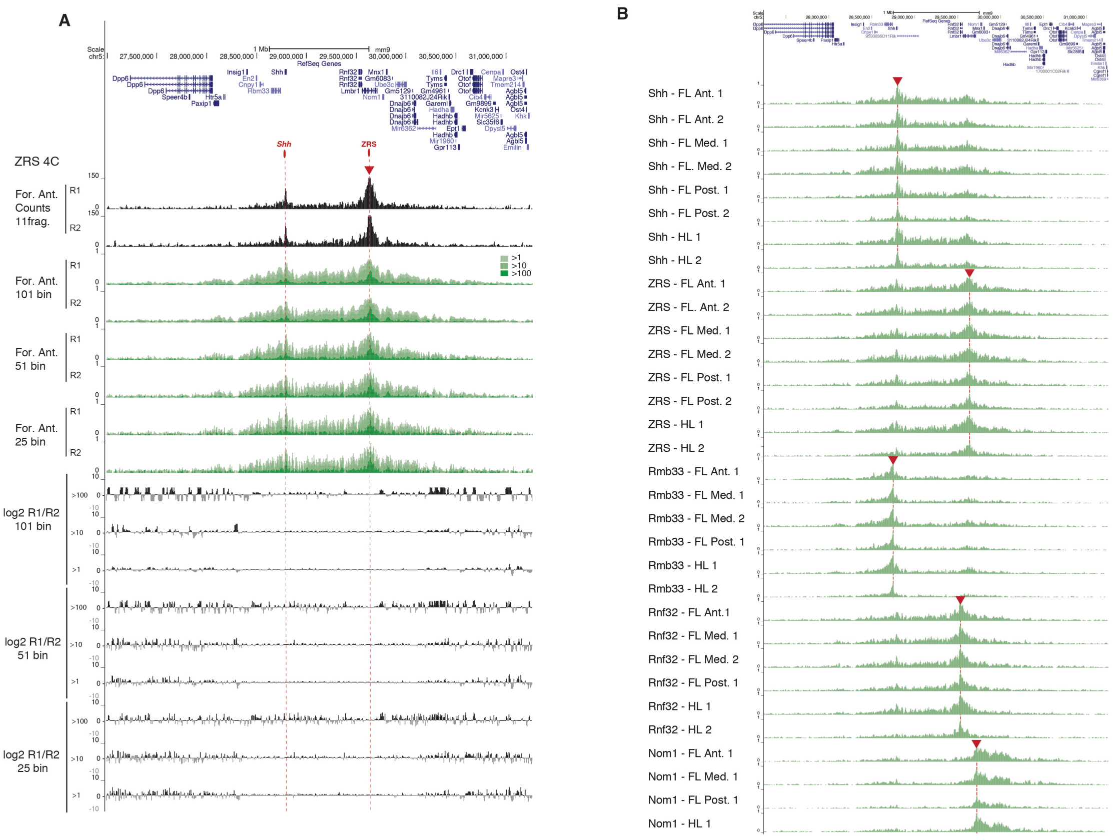

Figure S2, related to Figure 2. Topological organisation at the *Shh* locus in different limb compartments. (A) Biological replicates show very similar 4C profiles, regardless of threshold and bin size. Top panels show interaction profiles in the anterior forelimb obtained using different bin sizes (as labelled on y axis) and using different threshholds (Hit percent rate, intensity of green shows increasingly higher cut-off values), lower panel shows log2 ratios between replicates. (B) Comparison of 4C signals in different limb compartments and different viewpoints. The position of the viewpoints is shown by red arrows. FL: forelimb, HL: hindlimb, Ant: anterior, Post: posterior, Med: middle, 1 and 2 indicate replicates.

Figure S3 (related to Figure 3).

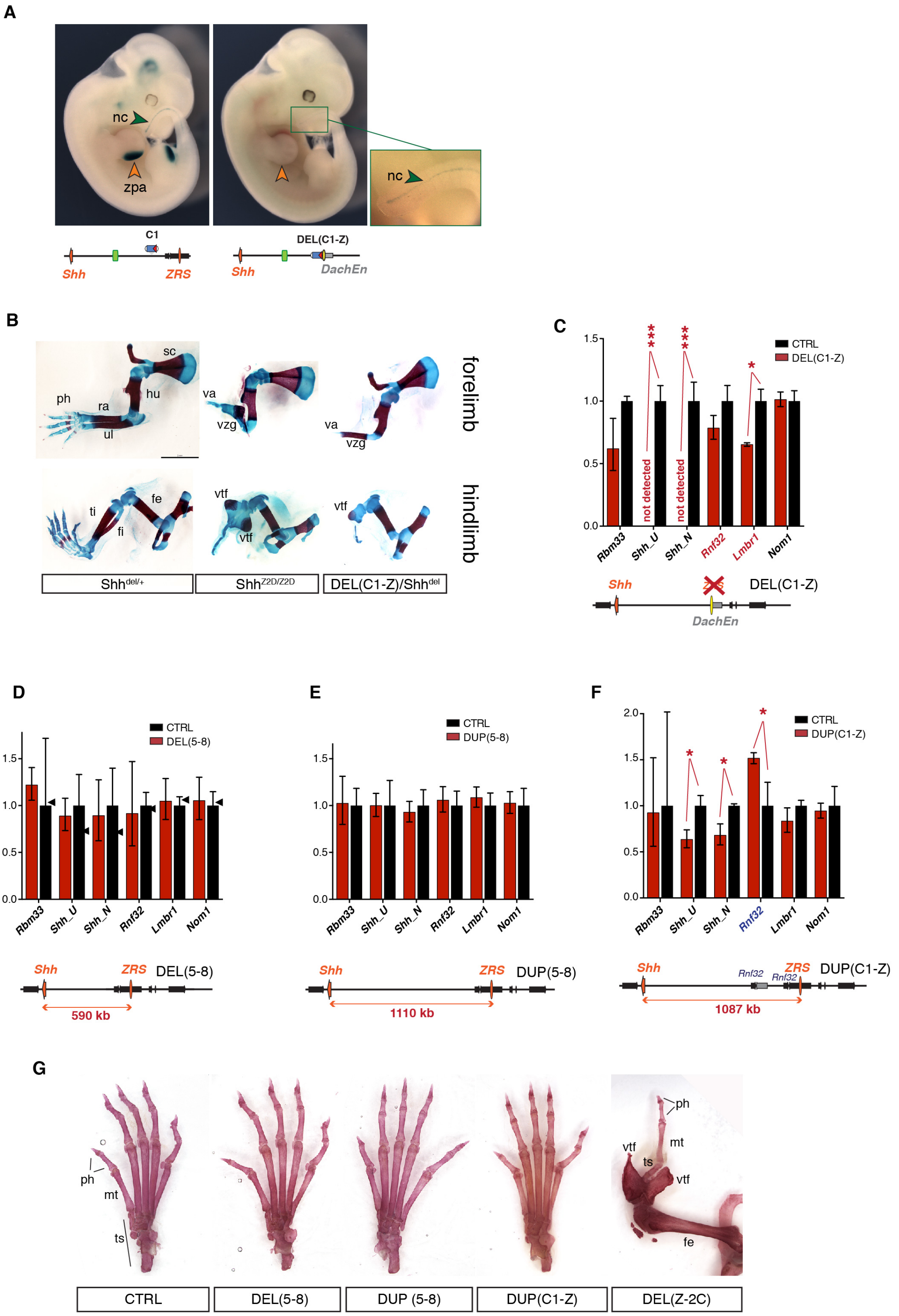

Figure S3, related to Figure 3. Changing distances within the *Shh* TAD.

(A) LacZ expression of the regulatory sensor at the SB-C1 position in the native locus (left) and in the context of DEL(C1-Z) allele (right). The orange arrowhead indicates expression (or lack thereof) in the ZPA, the green arrowhead expression in the notochord. A schematic outline of the corresponding locus is indicated below the pictures, with the ZRS shown as an orange oval, the region with notochord enhancers shown in green. In the DEL(C1-Z) allele the ZRS is replaced by the Dach enhancer (hs126 (Visel et al., 2007), yellow oval), which appeared to be essentially inactive when inserted at this position (Figure S3B and G). **(B)** Forelimb (top) and hindlimb (bottom) morphology of E18 embryos. Both homozygous Shh^{Z2D} embryos and DEL(C1-D)/Shh^{del} embryos have typical *Shh* limb loss-of-function phenotypes, but the phenotype is weaker in Shh^{Z2D} embryos, especially in the hindlimb where a fused tibia-fibula element remains. sc: scapula; hu: humerus; fz: fused zeugopod; ra: radius, ul: ulna, ph: phalanges, fe: femur, ti: tibia, fi: fibula, vzg: fused zeugopod, vtf: vestigial partially fused tibia-fibula, va: vestigial autopod. **(C-F)** Gene expression, assessed by RT-qPCR in DEL(C1-Z) (C), DEL(5-8) (D), DUP(5-8) (E) and DUP(C1-Z) (F) E11 forelimb buds. Homozygous mutant samples are in red (n=3), stage-matched wild-type samples from the same litters (n=3) are used as control, except for (D), where wild-type samples include embryos from separate litters (the arrows indicate the expression level in wild-type littermates of the mutants). **(G)** Foot (hindlimb) skeletons of adult mice with different rearranged alleles. Alleles are *in trans* of either Shh^{del} (for DUP(5-8), DUP(C1-Z), DEL(Z2C)) or of a ZRS replacement (Z2D allele, for CTRL and DEL(5-8)) ts: tarsus, mt: metatarsus, ph: phalanges, fe: femur, vtf: vestigial partially fused tibia-fibula.

Figure S4 (related to Figure 4).

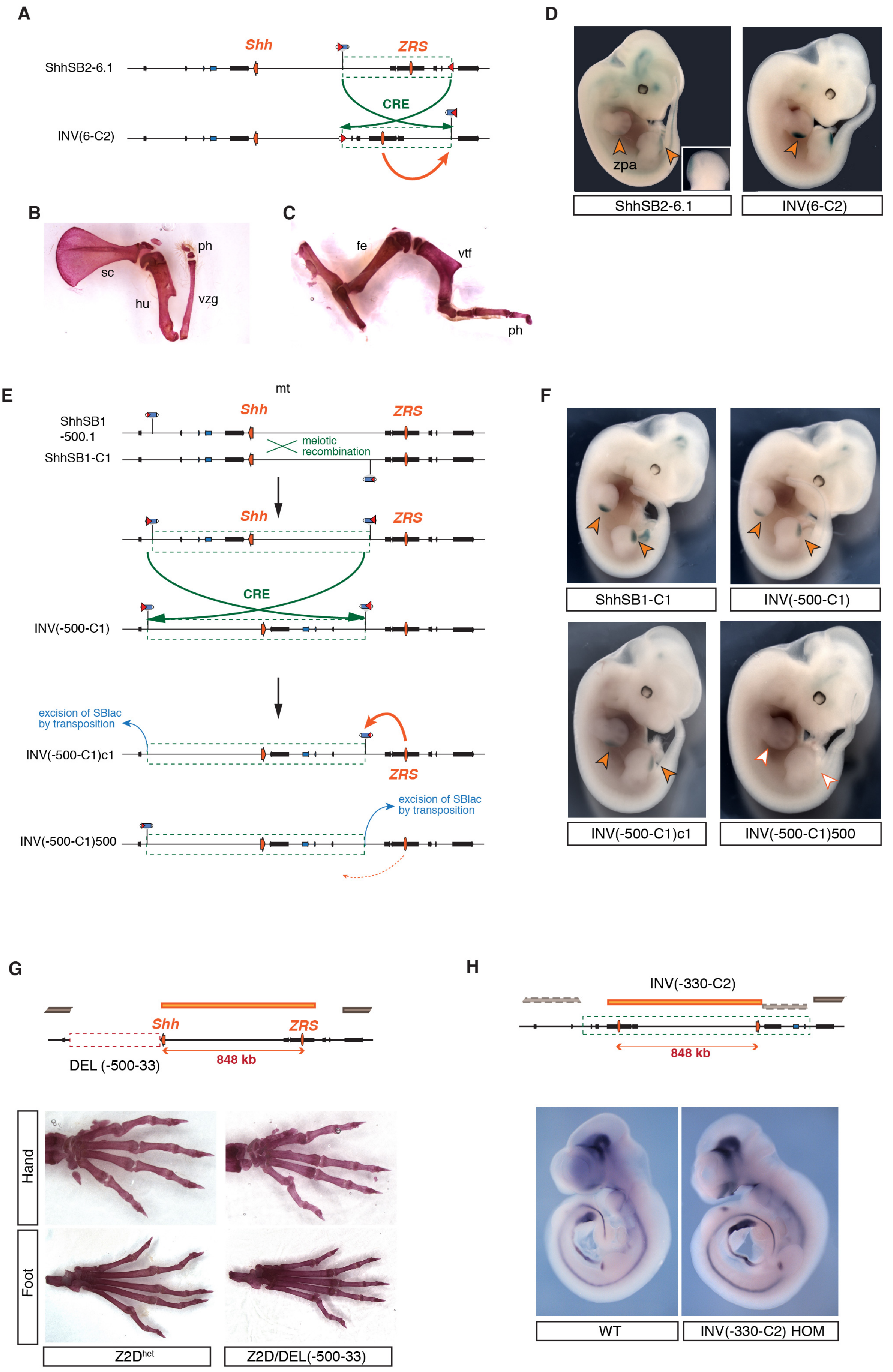

Figure S4, related to Figure 4. Consequences of TAD disrupting-alleles.

(A) Generation of the INV(6-C2) allele. The parental allele (ShhSB2-6.1) carried a *loxP* site at the SB-C2 location (telomeric of the ZRS) and a *loxP* site in opposite orientation in *cis* at the ShhSB2-6.1 position, allowing for *Cre*-mediated inversion of the DNA between the two *loxP* sites. **(B-C)** Adult fore- and hindlimb skeletons of INV(6-C2)/ Shh^{Z2D} mice, showing typical monodactyl and fused zeugopod phenotypes, characteristic for *Shh* loss-of-function in the limb. sc: scapula, hu: humerus, vzg: fused zeugopod, ph: phalange, fe: femur, vtf: vestigial tibia-fibula. **(D)** Expression of the regulatory sensor before (ShhSB1-6.1) and after (INV(6-C2)) inversion. The orange arrowhead shows expression in the ZPA. **(E)** Generation of the INV(-500-C1) allele. The starting point for the inversion were two insertions at positions ShhSB1-500kb (centromeric of *Shh*) and SB-C1 (between *Shh* and the ZRS). The two insertions had *loxP* sites in opposite orientations in *trans*, which were brought in *cis* through a meiotic recombination event during breeding. This intermediate allele was then subjected to *Cre*-mediated inversion. The obtained inversion allele (INV(-500-C1)) also contained two copies of the regulatory sensor at both ends of the inversion, which could be removed independently via remobilization of the transposon, giving rise to the INV(-500-C1)C1 and the INV(-500-C1)500 alleles. **(F)** *LacZ* expression in the ZPA of embryos with the INV(-500-C1) inversion allele. The parental ShhSB1-C1 allele is displayed as control, the ShhSB1-500kb insertion had no expression (not shown). The original inverted allele with two copies of the regulatory sensor at both breakpoints (INV(-500-C1)), and the two alleles after remobilization of one copy of the regulatory sensor (INV(-500-C1)C1 and INV(-500-C1)500) are shown. The orange arrowhead highlights expression in the ZPA, the white-and-red arrowhead lack thereof. **(G)** Hand and foot morphology of adult DEL(-500-33)/Shh^{Z2D} mice. The position of the deletion is indicated by the dashed red rectangle on the outline of the locus. The morphology of heterozygous Shh^{Z2D} mice is shown as control. **(H)** *Shh* expression in INV(-330-C2) embryos. The position of the inversion is indicated by the dashed green rectangle on the outline of the locus, orange and brown bars represent TADs. *Shh* expression was detected by whole-mount in situ hybridization in wild-type and homozygous INV(-330-C2) embryos at E10.5.

Figure S5 (related to Figure 5).

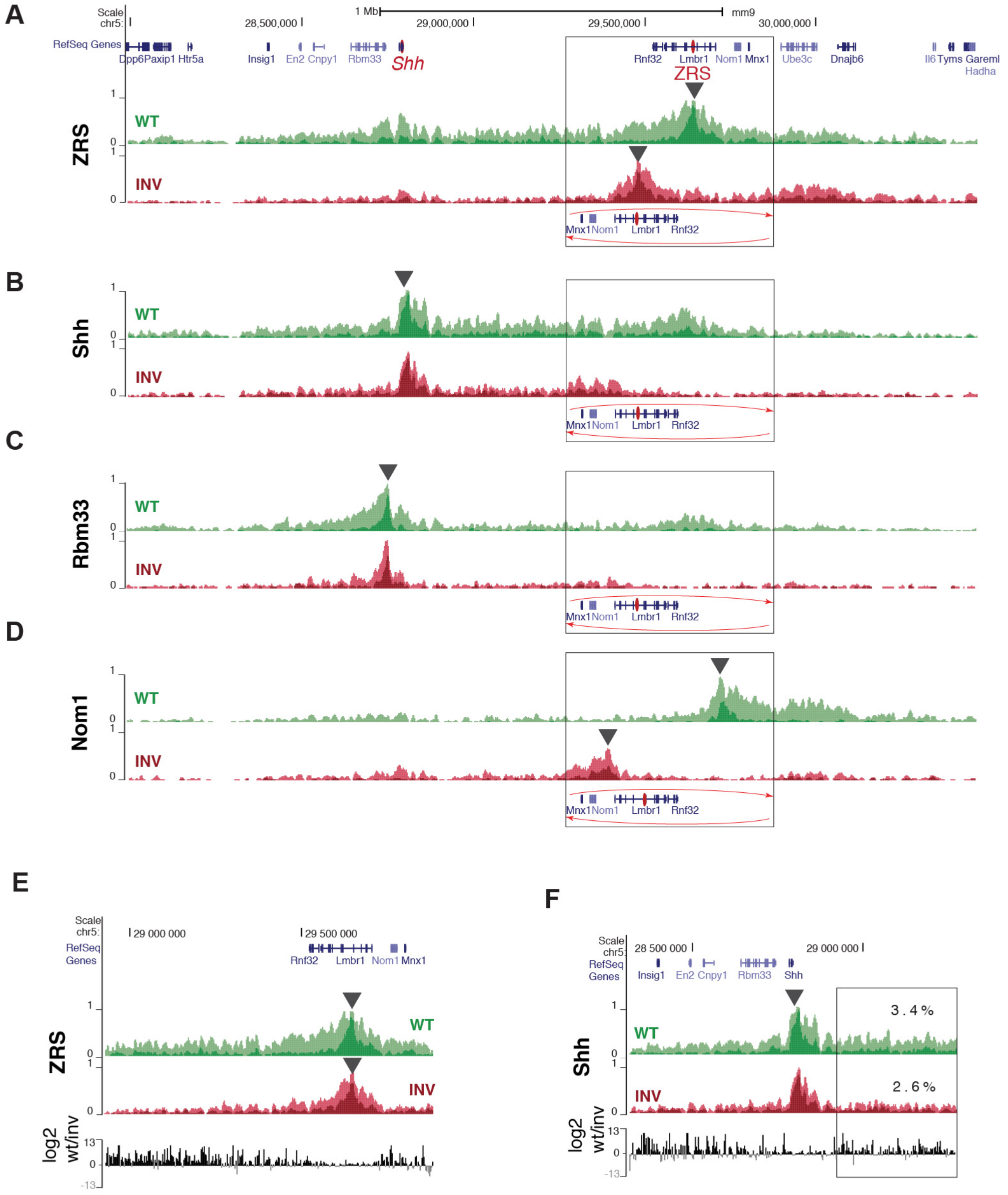

Figure S5, related to Figure 5. 4C profiles in INV(6-C2) alleles. (A-D) For each viewpoint, the normalized profiles obtained from WT (green) and INV (red) hindlimb samples are plotted on their respective genomic configurations (i.e. with an inversion of the [6-C2] genomic segment for INV). The position of the viewpoints is indicated with the black arrowhead. The inverted region is boxed, and the new position of the genes in the INV allele is depicted. **(E)** Comparison of the interaction profile of the ZRS between WT and INV in the inverted region (plotted with the same orientation) **(F)** Same comparison as in (E) for the interaction profile of *Shh* between WT and INV. The box delimitates the intra-TAD segment not affected by the inversion and the percentage of counts contained within it.

Figure S6 (related to Figure 6).

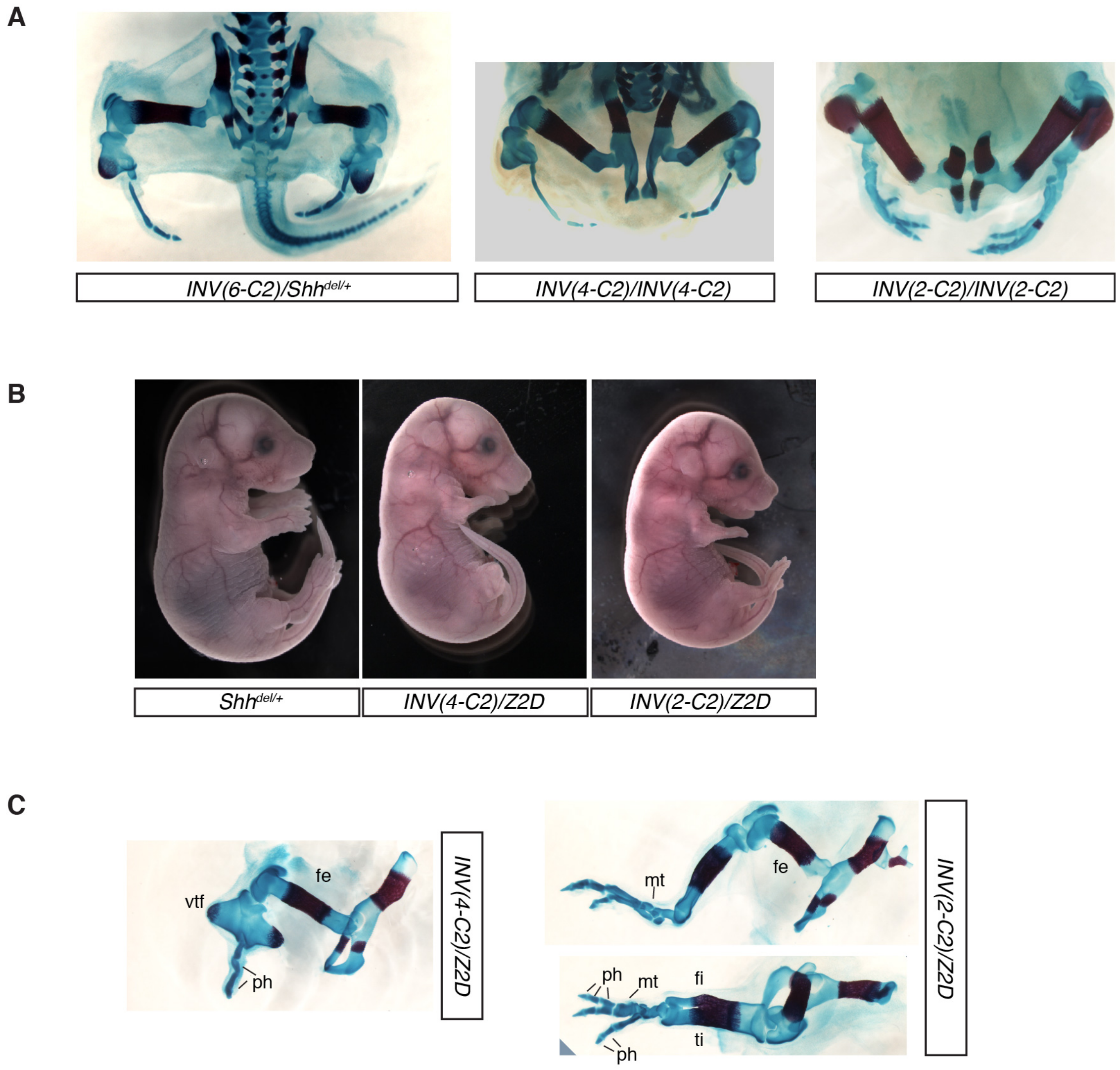

Figure S6, related to Figure 6. Phenotypic consequences of a TAD-breaking inversion series. (A) Hindlimb skeletons of E18 embryos carrying the INV(6-C2), INV(4-C2) and INV(2-C2) alleles. **(B)** Morphology of E13 embryos that are compound heterozygous for an inversion and the Z2D allele. Most of the gross phenotypic abnormalities seen in homozygous embryos or in embryos that were compound heterozygous over a *Shh* deletion are restored, with the exception of the truncated limb. A heterozygous *Shh*^{del} embryo at the same stage is also shown for comparison. **(C)** Hindlimb skeletons of E18 embryos that are compound heterozygous for INV(4-C2) and Z2D (a functional null allele of the ZRS) or for INV(2-C2) and Z2D. fe: femur, vtf: vestigial partially fused tibia-fibula, ph: phalange, mt: metatarsus, fi: fibula, ti: tibia.

Figure S7 (related to Figure 7).

A

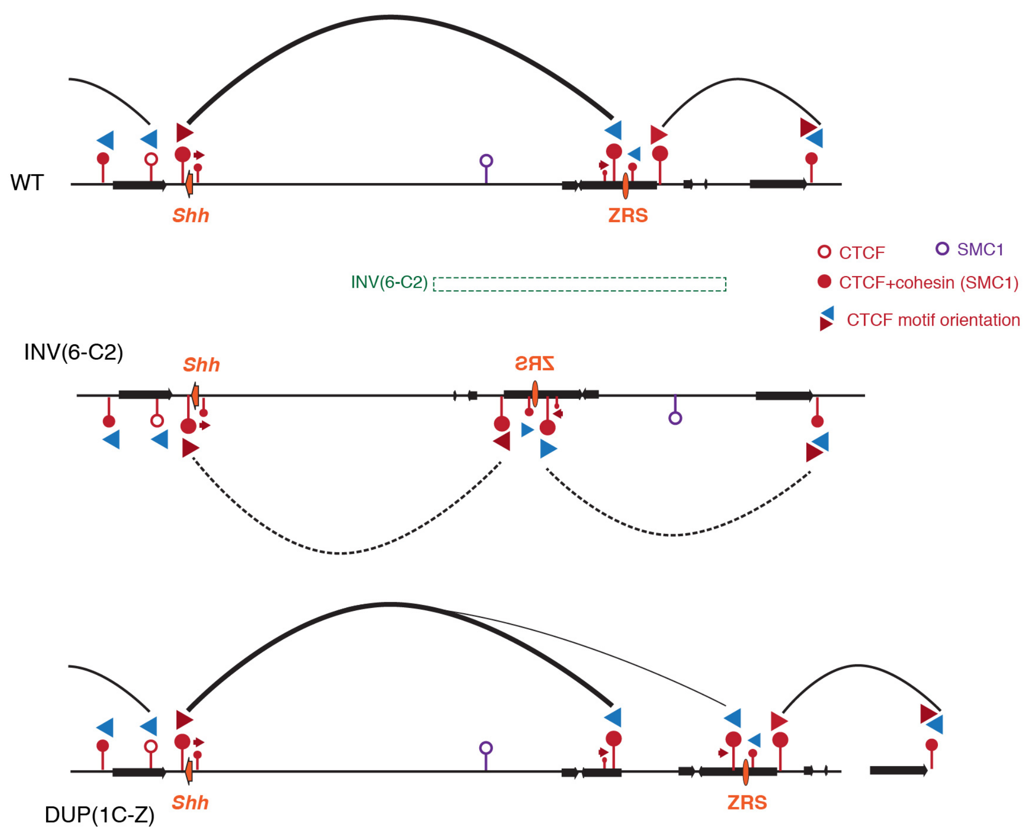

B

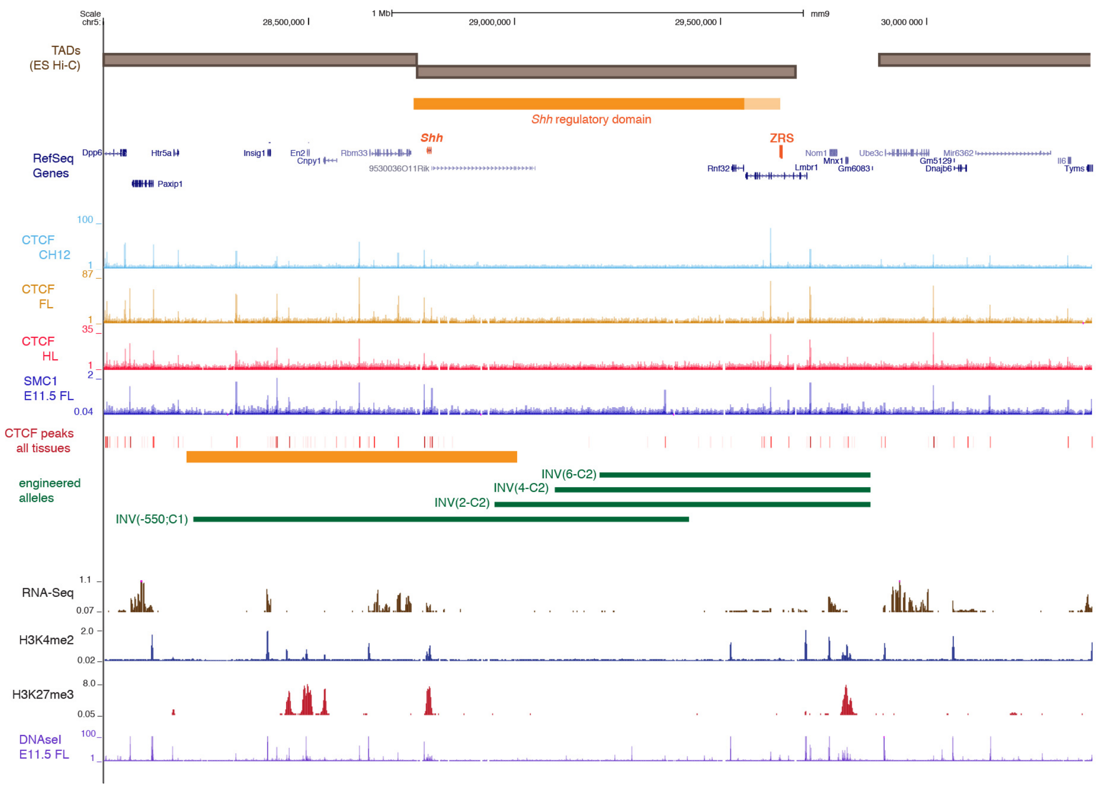

Figure S7, related to Figure7. Chromatin organisation around the *Shh*-ZRS locus. (A) Distribution and orientation of CTCF sites in the *Shh*-ZRS interval. Sites are shown by lollipops (size corresponding to ChIP-Seq peak height) and arrowheads indicate CTCF site relative orientation, based on the motif(s) identified below the peaks using JASPAR (Mathelier et al., 2016). Predicted loops by the CTCF-orientation model (de Wit et al., 2015; Guo et al., 2015; Rao et al., 2014) are shown by black lines. Our 4C data does not show evidence of of the isolated neighborhood predicted for INV(6-C2). In DUP(1C-Z), the formation of the normal CTCF-CTCF loop with the ZRS-proximal site should positioned the ZRS far outside of this loop **(B).** Chromatin map of the locus, indicating the different TADs (in brown) (Dixon et al., 2012) and the *Shh* regulatory domain (in orange) identified (this work and (Symmons et al., 2014)), as well as the distribution of CTCF and cohesin binding sites. ChIP-Seq tracks from CH12 cells (Shen et al., 2012) as well as from embryonic limbs (forelimb – FL, hindlimb HL) indicate CTCF and the cohesin subunit SMC1 (Demare et al., 2013). CTCF peaks from 19 tissues (Shen et al., 2012) were clustered on one track, with red intensity indicating the proportion of tissues where they were detected. Gene expression is shown by RNA-Seq (E10.5 forelimb (Cotney et al., 2012)). H3K4me2 (Demare et al., 2013), and DNAseI hypersensitivity (Mouse ENCODE Consortium et al., 2012) highlight accessible promoters and other regions in the locus. H3K27me3 track (Cotney et al., 2012) shows that repressive marks are only focused on developmentally-regulated gene promoters.

Table S1, related to Figure 1. List of insertions of the reporter sensor within and around the *Shh* locus. The position of each insertion is given as well as its expression pattern, assessed by LacZ staining, at specific stages of embryonic development.

as .xls file

Table S2, related to Figure 2. Comparison of responsiveness to the ZRS and genomic properties
For each insertion, the expression in the ZPA is determined in a semi-quantitative manner (0=nul, 1=weak, 2=strong). At each position, different genomic parameters have been measured. Proximity to ZRS, as determined by 4C (with normalized read-counts or hitpercent), accessibility (with DNAse I hypersensibility, DHS, data from ENCODE-WashU), density of various histones marks (H3K27ac, H3K27me3 – data from Cotney et al 2012, Demare et al. 2013), as well as distance to different classes of repeats (LINE1, SINE) are given.

as .xls file

Table S3, related to Experimental Procedures. List of primer sequences.

as .xls file

Table S4, related to Experimental Procedures. List and characteristics of 4C libraries

Supplemental Experimental Procedures

Transgenic mice

The founder **ShhSB1 and ShhSB2** mice were generated by homologous recombination in E14 ES cells. For the targeting construct we inserted a neomycin resistance cassette (under the control of a PGK promoter), flanked by FRT sites, and with (ShhSB2) or without (ShhSB1) an adjacent *loxP* site into a pSB8 plasmid. The pSB8 (Chen et al., 2013) plasmid consists of a *LacZ* reporter gene (the regulatory sensor, driven by a human β-globin minimal promoter) and a *lox*P site, which are cloned between the terminal inverted/direct repeats of the Sleeping Beauty transposon. The resulting SB-FRT-neomycin-FRT(-loxP) construct was inserted between homology arms that had previously been PCR amplified and cloned into a pSK Bluescript plasmid. The homology arms corresponded to chr5:29,410,542-29,413,901 and chr5:29,413,902-29,419,273 (NCBI37/mm9) for ShhSB1 and chr5: 29,848,614-29,854,582 and chr5:29,854,583-29,857,983 (NCBI37/mm9) for ShhSB2. Targeting constructs were validated by restriction digest and sequencing, and released using NotI.

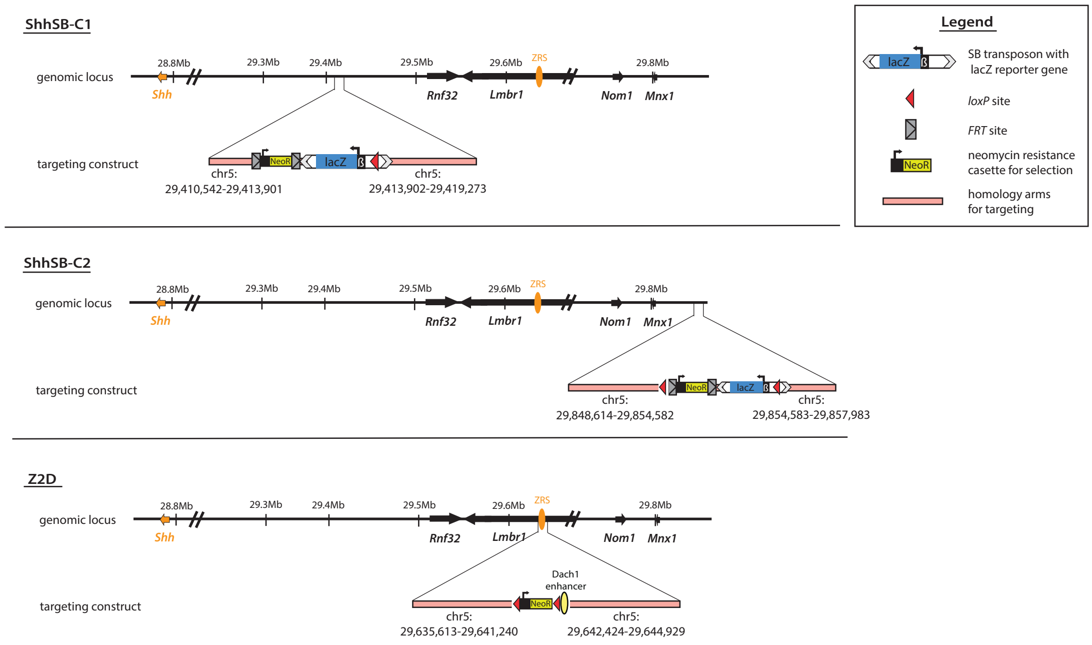

The **Z2D transgenic mouse line** was also generated by homologous recombination in E14 ES cells. In this line the ZRS enhancer (chr5:29,641,240-29,642,424) was substituted with a limb enhancer of the mouse *Dach1* gene (chr14: 97,485,490-97,486,674) and a single *loxP* site. To this end the *Dach1* enhancer and a *loxP* site were cloned between homology arms corresponding to chr5:29,635,613-29,641,241 and chr5:29,642,424-29,644,929 (NCBI37/mm9). The targeting construct was released using XbaI and XhoI. The *Dach1* enhancer was previously shown to drive expression of a reporter gene in the anterior part of the developing limb bud (element hs126 in the VISTA Enhancer Browser (Nobrega et al., 2003; Visel et al., 2007)).

Constructs were electroporated into E14 ES cells grown on inactivated DR3 feeders in ES cell medium: DMEM (Gibco, Cat.No.41965) with 15% FBS (PAN Biotech GmBH, Cat.No.2602), 1% L-glutamine (Gibco, Cat.No.25030-081), 1% penicillin/streptavidin (Gibco, Cat.No.15070-063), 1% Non-essential amino acids (Gibco, Cat.No.11140-050), 1% Sodium Pyruvate (Gibco, Cat.No.11360-070), 1% 2-mercaptoethanol (Sigma-Aldrich, Cat.No M7522) and and 1000 U/ml Leukaemia inhibitory factor (LIF, Chemicon, ESG1107). When E14 cells reached 70-80% confluence they were trypsinised, counted and 13 million cells were incubated in electroporation buffer at room temperature with approximately 12 mg of DNA for 5 minutes and electroporated (240 V, 500 μF, 4 mm cuvette).
Following electroporation, ES cells were then grown on feeder cells without selection for 3 days. On the third day gentamycin was added at a concentration of 200 μg/ml for 2 days, and subsequently at a concentration 250 μg/ml until the end of selection (~ 8-10 days).
After gentamycin selection 200-300 individual colonies per construct were picked and expanded. Integration of

the transgene was tested by long-range PCR and Southern blot. Positive ES clones were injected into mouse blastocysts, and chimeric males were bred to C57BL/6 for germ line transmission, which was confirmed by Southern blot and PCR. The neomycin selection cassette was subsequently removed by breeding to *hACTB*::FLPe transgenic animals. Following removal of the neomycin casette the SB transposon was remobilized and new insertions were mapped as described before (Ruf et al., 2011).

Deletions and duplications were engineered by TAMERE (Hérault et al., 1998; Wu et al., 2007), using *loxP* sites in the same orientation in *cis* (for SB-C2 derived deletions)) or in *trans*. Inversions were generated by *Cre*-mediated recombination using *loxP* sites in opposite orientation in *cis*. *loxP* sites in *cis* were obtained either using STRING (Spitz et al., 2005), i.e. breeding mice for meiotic recombination between 2 transposons in *trans,* or by using ShhSB2 remobs, where one *loxP* site remained fixed at the initial integration site (whereas the other was remobilized as transposon cargo). All rearrangements were verified by long-range PCR bridging the rearrangement breakpoint,

Long-range PCR
For long range PCR, DNA was prepared from ES cells or tails by adding 200µl lysis buffer (100mM Tris pH=8.5, 5mM EDTA, 0.2% SDS, 200mM NaCl), complemented with 1:100 dilution of 10mg/ml stock of ProteinaseK. Cells were lysed overnight at 65°C. The next day, material was precipitated with 140µl isopropanol at -20°C for 2h, centrifuged for 15min, the supernatant removed, and the DNA pellet washed with 70% ethanol. After additional centrifugation and removal of supernatant, the pellet was dried, and resuspended in 60µl TE. Long-range PCR was performed using the Expand Long Range dNTPack (Roche, Cat. No. 4829042001) with 0.5-1µl DNA as template. To test ES cells for integration of the SB insert via homologous recombination we used one primer anchored within the transgenic construct and one primer in the genomic region flanking the homology arms of the targeting construct. To test for deletions, duplications and inversions in mice we used primers for the genomic regions flanking the region. All primers are listed in Table S4.

Genotyping and genotyping strategies
For genotyping, tails or embryonic membranes were lysed overnight in lysis buffer (10 mM Tris-HCl pH 8, 50 mM KCl, 2 mM $MgCl_2$, 0.1 mg/ml gelatin, 0.45% NP-40 and 0.45% Tween-20) supplemented with proteinase K (100 µg/ml) and heat inactivated. 1µl of lysate was then used as template in PCR, using an in-house preparation of *Thermus aquaticus* (Taq) DNA polymerase (produced from a construct provided by the IGBMC, Strasbourg) and 10x PCR buffer (500mM Tris-HCl pH=9.5, 150mM $(NH_4)_2SO_4$ and 17.5mM $MgCl_2$). Genotypes were determined by PCR using transgene-specific primers for the transposon (SB8), the transposase (Prm), *HprtCre*, Shh^{del} and Shh^{Z2D}. SBlac insertions were genotyped with one primer specific to the given insertion site and one primer specific for the transposon. All rearrangements were routinely genotyped through the presence of novel primer combinations. Primers are listed in Table S3.

LacZ staining
E10.5, E11.5 or E12.5 mouse embryos were dissected in cold PBS, and fixed in PBS with 4% PFA on ice for 20, 30 or 40 min, respectively. Next, they were washed twice with ice-cold PBS and once at room temperature. Staining for β-galactosidase activity was performed overnight in a humid chamber at 37 °C. After staining, embryos were washed in PBS and stored at 4 °C.

Whole-mount RNA In situ hybridization
For whole-mount RNA *in situ* hybridization embryos were collected at E10.5 and E11.5, placed in 4% PFA for overnight fixation at 4°C. Yolk sacs were collected and processed for genotyping. The next day embryos were washed 3 times for 5 minutes in PBS containing 0.1% Tween-20 (PBS-T) and gradually dehydrated in 30%, 50%, 70% and 100% methanol containing PBS-T. Dehydrated embryos were stored at -20°C.

The *Shh* ISH probe was *in vitro* transcried from a plasmid from A. McMahon (linearized with HindIII, blunted with Klenow fragment and purified using QIAquick PCR Purification Kit (Qiagen, Cat. No. 28104)). 500ng DNA was used as template for DIG-labelled complementary probe synthesis with T3 polymerase using DIG RNA Labelling Kit (Roche). RNA probe was cleaned with GE Illustra ProbeQuant G50 kit (GE Healthcare Life Sciences, Cat.No. 28-9034-08), and eluted in 50µl RNase-free water. To remove any remaining DNA, the sample was treated with 1µl RNase-free DNase I.

Embryos were re-hydrated at 4°C in 70%, 50% and 30% methanol containing PBS-T and washed in PBS-T. After washing, embryos were bleached for 45 minutes to 1 hour in 6% H_2O_2 (diluted in PBS-T) and washed again in PBS- T. Bleached embryos were permeabilised by ProteinaseK treatment at room temperature for 7 (E10.5 embryos) or 12 (E11.5 embryos) minutes. ProteinaseK activity was stopped by washing with 2mg/ml glycine solution for 5 minutes on ice. The embryos were post-fixed at room temperature in 4% PFA for 20

minutes, and washed in PBS-T. Next, embryos were washed for 10 minutes in W1 (5X SSC pH4.5, 50% ionized Formamide, 1% SDS, 0.1% Tween- 20) at 65°C, and then pre-hybridised at 65°C in H2 (W1+ 5mg/ml Torula yeast RNA and 25µl of 100mg/ml heparin) for at least 2 hours. Embryos were incubated overnight at 65°C with the RNA probe in H2. The next day, embryos were washed for 3 times 30 minutes at 65°C in pre-heated W1, then also W2 (2X SSC pH=4.5, 50% ionized Formamide, 0.1% Tween-20), and finally once in W3 (2X SSC pH=4.5, 0.1% Tween-20). After equilibration at room temperature, embryos were washed in TBS-T (137mM NaCl, 20mM Tris, containing 1% Tween-20). For blocking, embryos were kept in blocking solution (TBS-T with 20µl fetal calf serum and 20µl of 100mg/ml BSA) for at least 2 hours, before adding DIG antibody (1:3000 dilution in blocking solution), and leaving them overnight at 4°C. On the third day, embryos were washed in TBS-T extensively, and kept in TBST overnight at 4°C.

The last day embryos were washed 3 times 10 minutes in NTMT (100mM Tris pH9.5, 100mM NaCl, 1% Tween-20), before staining with nitro-blue tetrazolium chloride and 5-bromo-4-chloro-3'-indolyphosphate p-toluidine salt (3.4µl/ml of 100mg/ml NBT and 3.5µL of 100mg/ml BCIP in NTMT) in a dark chamber. The staining reaction was stopped by washing in PBS after approx. 2-4 hours.

Alizarin red staining of adult skeletons

For alizarin red staining of bone adult mice (usually at 6 weeks or older) were euthanized by CO2 inhalation. After visual inspection of limbs, particularly checking for syn- and poldactyly, skin and internal organs were removed, and bodies were then fixed in 95% ethanol for 7 days. After fixation soft tissue was digested and bone was stained by incubation in 2% KOH with 0.001% alizarin red (Fluka) for 2-3 days. Remaining tissue was cleared with glycerol.

Alizarin red/alcian blue staining of embryonic skeleton

For skeletal preparation of late E17 embryos we used a modified protocol from (Wallin et al., 1994). Briefly, embryos were collected from pregnant females, and skin was removed by briefly incubating them in 60°C water. Internal organs were removed and embryos were fixed in 95% EtOH and 100% acetone for 1 week each. Next, they were transferred to alizarin red/alcian blue staining solution (1 volume 0.3% alcian blue (Fluka), 1 volume 0.1% alizarin red (Fluka), 1 volume acetic acid, 17 volumes 70% acetic acid) for 3 days. Finally, embryos were cleared by incubation in glycerol/1% KOH with an increasing ratio of glycerol.

Generating 4C libraries

For 4C libraries we collected the following embryonic mouse tissues:

- C57Bl/6 wild-type forelimbs (dissected into anterior, middle and posterior sections) and whole hindlimbs of E11.5 mouse embryos
- INV(6-C2)/del(-90kb-C2) whole forelimbs and hindlimbs. For this purpose heterozygous INV(6-C2) mice were crossed with heterozygous del(-90kb-ShhSB2) mice. Compound heterozygous embryos could phenotypically easily be distinguished from all other phenotypes due to their small size and truncated limbs.

All tissues were collected into PBS pH=7.4. Single cell suspensions were obtained by incubation in 1% trypsin at 37° C for 1 minute and rigorous pipetting with a blue P1000 tip. The cells were then immediately fixed with 2% formaldehyde in 10% FCS/PBS at room temperature for 10 minutes. Glycine was added to a final concentration of 0.1M and the samples were moved to ice to quench the cross-linking reaction. The cells were centrifuged at 8000 rpm for 8 minutes at 4°C, and the supernatant removed. Cells were lysed in 1ml cold lysis buffer (50mM Hepes pH=8, 150mM NaCl, 5mM EDTA, 0.5% NP-40 1.2% Triton X-100) for 10 minutes on ice. Successful lysis was verified by Methyl Green-Pyronin staining. Cell lysate was centrifuged at 8000 rpm at 4°C, the supernatant removed, and the pellet was frozen in liquid N2 until sufficient material was available.

Once sufficient tissue had been collected, cells were resuspended in 62.5 µl 10X NlaIII restriction buffer, placed at 37ºC and 15 µl 10% SDS was added. Cells were incubated for 1h at 37ºC, while shaking at 900rpm to remove non-crosslinked proteins. Subsequently, SDS was sequestered, by adding 150µl 10% Triton X-100 and incubated at 37ºC while shaking at 900 RPM. The sample was then digested by adding 400U (40 µl) NlaIII and incubation at 37ºC. After 4h and 8h another 400U of enzyme was added (total: 1200U NlaIII), adjusting the volume and buffer concentration as necessary, and the restriction digest was continued overnight , during the two latter digests. The next day we verified digestion efficiency by de-crosslinking and proteinaseK-treating samples taken before and after digest and running on an 0.6% agarose gel. If digest was not sufficient further enzyme was added for another 4h.

Following sufficient digestion, enzyme was inactivated by incubating 20 minutes at 65°C and samples were transferred to a 50ml falcon tube. Samples were ligated in a 7ml volume using 700µl 10X ligation buffer (0.3M Tris-HCl pH=7.8, 0.1M $MgCl_2$, 1.54% DTT, 0.5% ATP) and 50U T4 DNA ligase and incubated at 16°C overnight. We determined ligation efficiency by de-crosslinking and proteinaseK-treating 100µl of ligation reaction and running on an 0.6% agarose gel. If further ligation was necessary, we added fresh ATP and

continued ligation for 4-6h.
Once ligation was complete we de-crosslinked the sample overnight at 65ºC in the presence of 30µl Proteinase K (10mg/ml). The next morning, we added 30µl RNAse A (10mg/ml) and incubated the sample for 45 min at 37ºC. We then extracted DNA by adding 7ml phenol-chloroform and precipitating DNA using 7ml water, 1.5 ml 2M NaAcetate pH 5.6, 7 µl 20mg/ml glycogen and 35 ml 100% ethanol. The resulting pellet was dissolved in 150µl Tris pH 7.5 at 37ºC.
The obtained 3C library was then subjected to a second round of overnight digestion using 60U DpnII in a 500µl volume. The next day we verified digestion efficiency and subsequently inactivated the enzyme by incubating the sample at 65ºC for 25 minutes. The sample was then subjected to phenol-chloroform extraction, resuspended in 100µl water and ligated overnight with 100U T4 DNA ligase in a final volume of 14.3ml. The next day the DNA was first phenol-chloroform extracted and then purified using the QIAquick PCR purification kit, using 3 columns per sample, eluting with 50µl 10mM Tris per column.
4C primers for the region of interest were selected from the Tanay lab 4C primer database (van de Werken et al., 2012), further tested for off-targets using BiSearch (Arányi et al., 2006) and only primers with <50 hits were kept. For quality assessment of primers we tested them on 25, 50, 100 and 200ng of 4C template and kept only those with reproducible banding pattern at all concentrations. The final list of 4C primers is included in the Table S4. Using these primers, we set up PCR reactions for sequencing with primers containing barcodes and the Solexa sequencing adapter at their 5' end.
For PCRs we used Expand Long Template system from Roche, using either 100 or 200ng of the 4C library as template and setting up 16 PCR reactions in parallel for each primer. PCR products were purified using Roche HighPure PCR product purification kit, adding an additional 1 min spin after the last wash step to remove all residual ethanol. The samples were eluted in 50µl 10 mM Tris pH=8.0, pooled and 1 µg each of approximately 40 PCR products were pooled to achieve a balanced base composition at the first 4 bases.

Supplementary Table S3 (related to Experimental Procedures) – Primer sequences
as .xls file

Supplementary Table S4 (related to Experimental Procedure) – List of 4C libraries: summary information and quality controls.

Library	Raw Reads	Low Quality Reads	Mapped Reads	Mapping Ratio	Trans Counts	Cis Counts	Cis Ratio	Trans Counts	Cis Counts (-1Mb vwp)	Cis Ratio (-1Mb vwp)
**Shh_28804_FA_1**	1730807	0	1630977	0.94	693294	937683	0.57	693294	747864	0.21
**Shh_28804_FA_2**	1914379	0	1775919	0.93	965390	810529	0.46	965390	560664	0.21
**Shh_28804_FM_1**	2540591	0	2403406	0.95	973660	1429746	0.59	973660	1129771	0.24
**Shh_28804_FM_2**	1981742	0	1855936	0.94	656294	1199642	0.65	656294	881489	0.33
**Shh_28804_FP_1**	1939550	0	1754581	0.90	858547	896034	0.51	858547	680800	0.20
**Shh_28804_FP_2**	1380275	0	1305467	0.95	894833	410634	0.31	894833	260706	0.14
**Shh_28804_H_1**	1889315	0	1809985	0.96	730969	1079016	0.60	730969	867404	0.22
**Shh_28804_H_2**	1968711	0	1795179	0.91	1052237	742942	0.41	1052237	585991	0.13
**Shh_28804_invF_1**	2598357	0	2470169	0.95	1242232	1227937	0.50	1242232	990738	0.16
**Shh_28804_invH_1**	1745224	0	1607727	0.92	816800	790927	0.49	816800	559355	0.22
**Shh_28806_FA_1**	1395209	0	1330831	0.95	549917	780914	0.59	549917	582472	0.27
**Shh_28806_FP_1**	1576901	0	1498786	0.95	695601	803185	0.54	695601	579363	0.24
**Shh_28806_H_1**	1302506	0	1247398	0.96	493281	754117	0.60	493281	590136	0.25
**Shh_28806_H_2**	1531435	0	1457472	0.95	466535	990937	0.68	466535	782445	0.31
**ZRS_FA_1**	2113844	0	1991871	0.94	820855	1171016	0.59	820855	865876	0.27
**ZRS_FA_2**	2307615	0	2171679	0.94	978616	1193063	0.55	978616	803521	0.28
**ZRS_FM_1**	1179046	0	1083418	0.92	411309	672109	0.62	411309	474574	0.32
**ZRS_FM_2**	2056175	0	1920819	0.93	600006	1320813	0.69	600006	933723	0.39
**ZRS_FP_1**	2483997	0	2310022	0.93	1010325	1299697	0.56	1010325	965947	0.25
**ZRS_FP_2**	1923942	0	1773280	0.92	922651	850629	0.48	922651	608316	0.21
**ZRS_H_1**	2210918	0	2045358	0.93	843989	1201369	0.59	843989	906189	0.26
**ZRS_H_2**	2800697	0	2575402	0.92	1426290	1149112	0.45	1426290	848110	0.17
**ZRS_invF_1**	2688247	0	2433454	0.91	1477965	955489	0.39	1477965	585105	0.20
**ZRS_invH_1**	2366582	0	2201760	0.93	1145465	1056295	0.48	1145465	674751	0.25
**Rbm33_FA_1**	3663217	0	3521871	0.96	689794	2832077	0.80	689794	2630637	0.23
**Rbm33_FM_1**	3469042	0	3338762	0.96	627580	2711182	0.81	627580	2510876	0.24
**Rbm33_FM_2**	3620884	0	3480692	0.96	560906	2919786	0.84	560906	2638088	0.33
**Rbm33_FP_1**	3575961	0	3398238	0.95	727884	2670354	0.79	727884	2479788	0.21
**Rbm33_H_1**	3858835	0	3717274	0.96	635881	3081393	0.83	635881	2863760	0.25
**Rbm33_H_2**	3777146	0	3587211	0.95	923720	2663491	0.74	923720	2498848	0.15
**Rbm33_invF_1**	1354644	0	1309030	0.97	524548	784482	0.60	524548	720174	0.11
**Rbm33_invH_1**	1155364	0	1105181	0.96	377230	727951	0.66	377230	646859	0.18
**Nom1_FA_1**	782770	0	739755	0.95	371350	368405	0.50	371350	238591	0.26
**Nom1_FM_1**	585510	0	532208	0.91	249894	282314	0.53	249894	179185	0.29
**Nom1_FP_1**	408472	0	378472	0.93	203261	175211	0.46	203261	105908	0.25
**Nom1_H_1**	868031	0	818688	0.94	371599	447089	0.55	371599	316557	0.26
**Nom1_invF_1**	341516	0	322357	0.94	213545	108812	0.34	213545	58010	0.19
**Nom1_invH_1**	802372	0	745905	0.93	406257	339648	0.46	406257	188412	0.27
**Rnf32_FA_1**	1947975	0	1882565	0.97	650825	1231740	0.65	650825	985970	0.27
**Rnf32_FM_1**	2132872	0	2075854	0.97	681974	1393880	0.67	681974	1098263	0.30
**Rnf32_FM_2**	1370276	0	1295654	0.95	421023	874631	0.68	421023	637071	0.36
**Rnf32_FP_1**	1960298	0	1891582	0.96	714008	1177574	0.62	714008	930819	0.26
**Rnf32_H_1**	1880914	0	1833459	0.97	637401	1196058	0.65	637401	985745	0.25
**Rnf32_H_2**	2262878	0	2141327	0.95	892810	1248517	0.58	892810	1055835	0.18

Supplemental References.

Arányi, T., Váradi, A., Simon, I., and Tusnády, G.E. (2006). The BiSearch web server. BMC Bioinformatics *7*, 431.

Chen, C.-K., Symmons, O., Uslu, V.V., Tsujimura, T., Ruf, S., Smedley, D., and Spitz, F. (2013). TRACER: a resource to study the regulatory architecture of the mouse genome. BMC Genomics *14*, 215.

Cotney, J., Leng, J., Oh, S., Demare, L.E., Reilly, S.K., Gerstein, M.B., and Noonan, J.P. (2012). Chromatin state signatures associated with tissue-specific gene expression and enhancer activity in the embryonic limb. Genome Res *22*, 1069–1080.

Demare, L.E., Leng, J., Cotney, J., Reilly, S.K., Yin, J., Sarro, R., and Noonan, J.P. (2013). The genomic landscape of cohesin-associated chromatin interactions. Genome Res *23*, 1224–1234.

Mathelier, A., Fornes, O., Arenillas, D.J., Chen, C.-Y., Denay, G., Lee, J., Shi, W., Shyr, C., Tan, G., Worsley-Hunt, R., et al. (2016). JASPAR 2016: a major expansion and update of the open-access database of transcription factor binding profiles. Nucleic Acids Res *44*, D110–D115.

Mouse ENCODE Consortium, Stamatoyannopoulos, J.A., Snyder, M., Hardison, R., Ren, B., Gingeras, T., Gilbert, D.M., Groudine, M., Bender, M., Rajinder, K., et al. (2012). An encyclopedia of mouse DNA elements (Mouse ENCODE). Genome Biology *13*, 418.

Nobrega, M.A., Ovcharenko, I., Afzal, V., and Rubin, E.M. (2003). Scanning human gene deserts for long-range enhancers. Science *302*, 413.

Visel, A., Minovitsky, S., Dubchak, I., and Pennacchio, L.A. (2007). VISTA Enhancer Browser--a database of tissue-specific human enhancers. Nucleic Acids Res *35*, D88–D92.

Wallin, J., Wilting, J., Koseki, H., Fritsch, R., Christ, B., and Balling, R. (1994). The role of Pax-1 in axial skeleton development. Development *120*, 1109–1121.

Wu, S., Ying, G., Wu, Q., and Capecchi, M.R. (2007). Toward simpler and faster genome-wide mutagenesis in mice. Nat Genet *39*, 922–930.
